# Game analysis of green finance assisting enterprises in carbon reduction under the participation of four parties

**DOI:** 10.3389/fpubh.2025.1641134

**Published:** 2025-09-01

**Authors:** Jinlong Wang, Xiangbin Liu, Hongcheng Duan, Lizhi Wang

**Affiliations:** ^1^School of Finance, Harbin University of Commerce, Harbin, China; ^2^Accounting School, Harbin Finance University, Harbin, China

**Keywords:** green finance, carbon reduction, four party entity, evolutionary game, green transformation

## Abstract

Green finance is an important measure to promote industries' green and low-carbon development, which is of great significance for achieving high-quality economic development. This article constructs a four-party evolutionary game model of “government regulatory departments, banks, non-bank financial institutions, and high-carbon enterprises,” exploring the strategic choices and evolutionary trends of the four parties in the process of green finance and promoting the green development of high-carbon enterprises. Research has shown that: (1) Government regulatory agencies should establish a sound reward and punishment mechanism. Increasing subsidies and punishment will promote the system to evolve to an ideal stable state. Still, there is a threshold for reward and punishment intensity, and its effect shows diminishing marginal benefits. (2) When banks with information advantages have a high initial willingness, they will transmit green concepts through signal effects to encourage non-bank financial institutions to actively enter the green finance market, filling the existing green credit funding supply gap and helping enterprises reduce carbon emissions. (3) The high transformation costs faced by high-carbon enterprises are still the main reason for their choice of excessive emissions. The strategic choice of enterprises mainly depends on their transformational willingness. In addition, according to the theory of willingness behavior, there is a linkage effect and mutual influence between government regulatory departments and high-carbon enterprises in their strategic choices. For the carbon reduction behavior of enterprises, whether financial institutions provide sufficient green funding support is not a determining factor, but it will also play an important role.

## 1 Introduction

With the acceleration of global industrialization, issues such as air pollution and water pollution caused by corporate carbon emissions have become significant factors affecting public health. Studies have shown that long-term exposure to high concentrations of pollutants (such as PM2.5 and sulfur dioxide) can significantly increase the incidence of respiratory diseases, cardiovascular diseases, and cancer (1, 2). Meanwhile, as the core institution addressing health challenges, the operational activities of the healthcare system (such as energy consumption and waste generation) also have a non-negligible impact on the environment. For instance, improper disposal of medical waste generated by healthcare institutions may cause secondary pollution, further threatening ecosystems and human health. Therefore, exploring the bidirectional interaction mechanism between the environment and the healthcare sector has become a key proposition for achieving sustainable development. Driven by the tide of carbon neutrality, green finance plays a crucial role in building a Chinese path to modernization in which people and nature coexist harmoniously (3). Since the proposal of the “dual carbon” goal, green transformation and green technology innovation have increasingly become a hot topic of concern (4). In March 2021, China's 14th Five-Year Plan clearly stated the need to vigorously develop green technology innovation and promote transforming critical industries and essential fields. The report of the 20th National Congress of the Communist Party of China has put forward precise requirements for “accelerating the green transformation of development methods,” emphasizing that “promoting green and low-carbon economic and social development is a key link to achieving high-quality development.” In December 2022, the National Development and Reform Commission and the Ministry of Science and Technology jointly issued the Implementation Plan for Further Improving the Market-Oriented Green Technology Innovation System (2023–2025), which clearly stated the need to improve further the relevant work of the market-oriented green technology innovation system, providing technological solid support for promoting the green transformation of development modes and promoting high-quality development. From this, it can be seen that the green and low-carbon transformation has risen to the national strategic level, pointing out the direction for the future development of high-carbon enterprises while also posing more significant challenges (5, 6). However, due to the dual constraints of environmental regulations and limited resources, achieving green transformation is a practical problem faced by developing high-carbon enterprises (7). Green finance, as a market-oriented tool for environmental governance, can effectively reduce corporate carbon emissions by guiding capital toward low-carbon industries, thereby indirectly improving environmental quality and reducing public health risks. From this, the development of green finance is of great significance in solving the financial constraints faced by the transformation of high-carbon enterprises. Currently, banks are the main executors of green finance in China. However, the proportion of green credit to various loans is only 9–10%, and the massive gap in green funds makes the role of green finance very limited (8). Therefore, it is necessary to attract more financial entities to enter the green finance market and further promote the development of the green finance market by enriching and innovating green finance products. However, existing research has primarily focused on the direct impact of green finance on enterprises or industries, with less exploration into how it indirectly influences the healthcare system through environmental improvement, or how the healthcare system can contribute to environmental governance through green practices. This paper takes green finance's role in supporting corporate carbon reduction as a starting point, and through a four-party evolutionary game model, analyzes the dynamic strategic interactions among the government, financial institutions, high-carbon enterprises, and the public (including healthcare practitioners and patients) in carbon reduction, revealing the transmission mechanism of green finance in the bidirectional exchange between the environment and healthcare.

## 2 Literature review

The essence of green finance is to combine finance with environmental development and use economic means to promote high-quality economic development. The construction of the current green finance system is the focus of various scholars' research on green finance (9–11). The green finance system mainly includes green credit, securities, insurance, funds, etc. Currently, green finance in China is still primarily focused on green credit. Scholars' research on green finance mainly focuses on using green credit policies as a quasi-natural experiment to study their impact on enterprise green total factor productivity (12–14), green technology innovation (15–19), and green governance performance (20–22). In addition, there is also research on the effect of financial technology (23–25), blockchain technology (26), and other technologies on the development of green finance.

In addition to research on green credit, research on promoting high-quality economic development through green bonds (27), green investment (28, 29), green insurance (30, 31), and green funds (32, 33) in the green finance system has also received widespread attention from scholars. Previous studies have empirically tested the economic effects of green finance at the macro level, verifying its positive role in economic development. However, there has been no detailed characterization of the strategic choices of various microenterprises in the development of green finance. In addition, the current focus is mainly on empirical analysis, with less use of mathematical models to depict and analyze the evolution trend of micro-level strategy choices of various participating entities in promoting corporate carbon reduction in green finance.

Evolutionary game models can describe how game players achieve ideal states through continuous learning and adjustment of their strategic choices. Selection and mutation are its main characteristics, just as green finance is a sudden change in financial models compared to traditional finance. At the same time, the trade-off between green and conventional finance is also a choice for financial institutions. Therefore, introducing evolutionary games into the analysis of green finance-related issues has specific applicability. Existing research has also introduced evolutionary game models into the study of green finance. Firstly, some scholars analyze the relevant topics of strategy selection among different participating entities in the process of promoting green development of enterprises through constructing a tripartite model consisting of banks, non-bank financial institutions, enterprises (34), government regulatory departments, banks, enterprises (35), constructing an evolutionary game model that includes government financial institutions, enterprises, and consumers (36). Some scholars have also analyzed the construction of a green supply chain financial system and its essential significance for promoting the green and low-carbon transformation of enterprises by constructing a tripartite evolutionary game model that includes small and medium-sized enterprises, core enterprises, and financial institutions (37), or a four-party evolutionary game model that provides for green small and medium-sized enterprises, core enterprises, financial institutions, and government (38). Previous studies have mainly analyzed banks as gaming entities, but the current green funding gap is increasing yearly. Therefore, it is necessary to mobilize other financial entities within the financial system to participate actively in the green finance market, expand green finance channels to compensate for the shortage of green funds, and promote the green transformation of enterprises. In addition, due to the low enthusiasm of financial institutions for implementing green credit and the high cost of transformation, the willingness of enterprises to undergo green transformation is generally low. Therefore, regulatory authorities must establish a sound reward and punishment mechanism to mobilize the enthusiasm of relevant entities and promote high-quality economic development.

This article is based on the macroeconomic regulation environment led by government regulatory departments, dividing financial institutions into banks and non-bank financial institutions. By constructing a four-party evolutionary game model of government regulatory departments, bank financial institutions, non-bank financial institutions, and high-carbon enterprises, the evolutionary trend of various parties' strategic choices in promoting enterprise carbon reduction through green finance is depicted from a dynamic perspective. Numerical simulation analysis is used to explore how the changes in various parameters affect the evolution trend of the strategic choices of each entity and how measures are ultimately taken to achieve the ideal state of the evolutionary system. This provides guidance and a reference for promoting the realization of China's “dual carbon” goals.

## 3 Problem description and model construction

### 3.1 Problem description

Driven by the “dual carbon” goal, promoting green transformation in critical industries and areas has become the primary goal of achieving high-quality development. High-carbon industries, represented by petrochemicals, steel, etc. have become essential for green transformation development (39, 40). However, the high transformation cost has generally led to a lower willingness of high-carbon enterprises to undergo green transformation. The development of green finance has allowed high-carbon enterprises to reduce transformation costs. However, the current willingness of banks and financial institutions to implement green loans is relatively low, and the gap in the green capital market is also significant. Therefore, attracting more financial entities to enter the green finance market is necessary to expand the supply channels of green funds. In response to the above issues, this article intends to use the evolutionary game method to place government regulatory departments, banks, non-bank financial institutions, and high-carbon enterprises under the same analytical framework. Based on the background of government macroeconomic regulation, financial institutions are divided into banks and non-bank financial institutions to systematically analyze the evolution process of various stakeholders' strategic choices in green finance promoting carbon reduction in high-carbon enterprises. This article mainly explores the following questions: (1) Under the existing green finance system, can non-financial institution finance entering the green credit market make up for the existing gap in the supply of green credit funds? (2) Under the background of the “dual carbon” goal, can green finance help high-carbon enterprises reduce emissions to achieve sustainable economic and environmental development? (3) By combining signal effect theory, willingness behavior theory, and evolutionary game theory, we can deeply analyze how changes in behavioral strategies among different entities in the green finance system affect the strategic choices of other entities and generate linkage effects.

**Figure 1:**
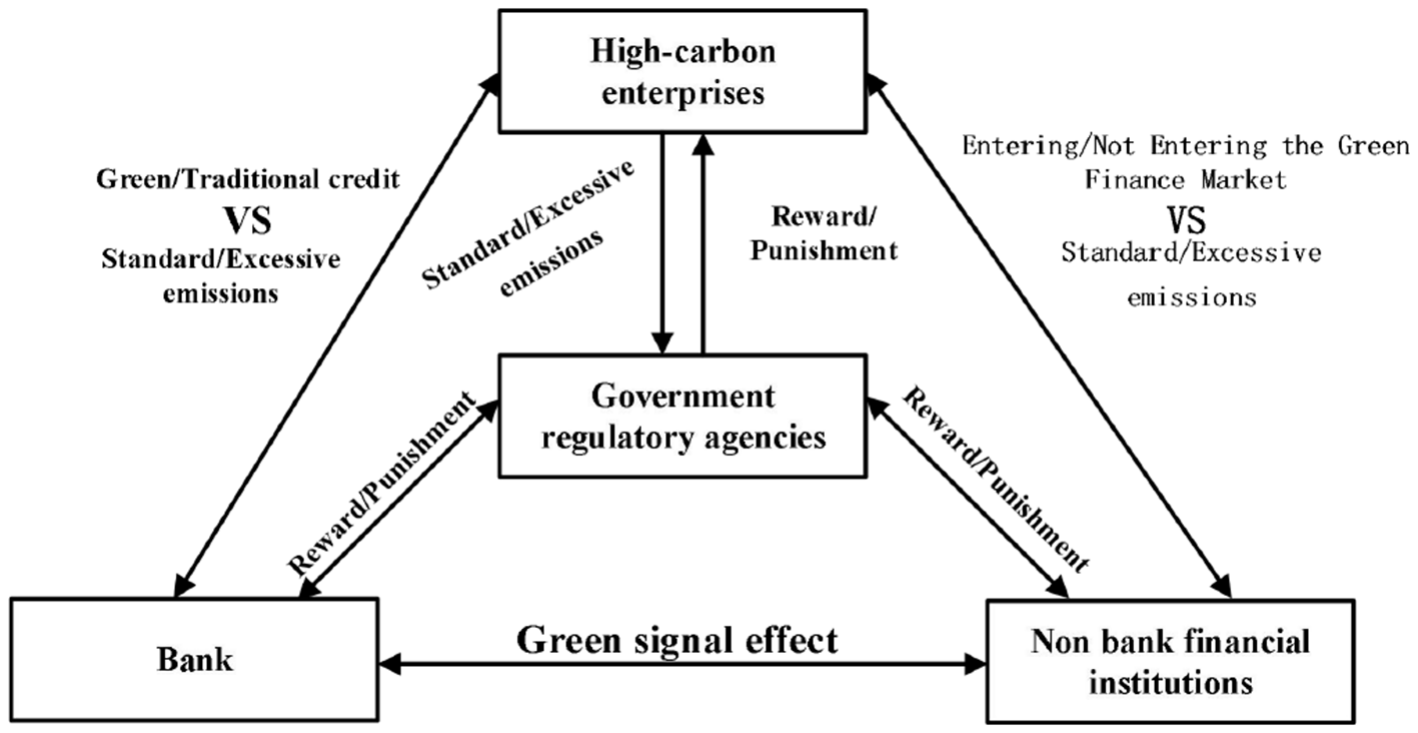
A logic diagram of decision-making among the four parties.

This study constructs the decision-making logic relationship between government regulatory departments, banks, non-bank financial institutions, and high-carbon enterprises, as shown in Figure 1. The graph shows that government regulatory agencies are at the core of regulation, and they play a macroeconomic regulatory role in promoting green development by establishing a sound reward and punishment mechanism. Green concept transmission between banks and non-bank financial institutions has a signal effect (41, 42). When banks are highly willing to implement green finance policies, they will transmit green development concepts within the economic system through the signal effect, thereby attracting non-bank financial institutions to actively enter the green finance market and jointly promote the development of the green finance market. The green signal effect transmission between banks and non-bank financial institutions is essentially achieved through mechanisms such as information sharing, product linkage, and reputation transmission, which transform the environmental performance of enterprises (such as carbon emission standards and ESG rating improvement) into positive feedback in the financial market, forming a closed loop of “green signal resource aggregation low-carbon transformation.” The specific methods include: (1) Information layer: Build a unified and transparent green data system to solve the problem of “signal distortion”; (2) Product layer: Design cross market and cross term combination tools to meet diversified green financing needs; (3) Market layer: Connect the credit, bond, equity, and derivative markets to form a closed loop of funds; (4) Policy level: Strengthen institutional incentives through regulatory assessments and policy tool coverage. High-carbon enterprises are limited by government regulatory agencies' environmental pressure and the high transformation cost. They will weigh between exceeding or meeting emissions standards. At this time, the reward and punishment mechanisms of regulatory agencies and the supply of green funds by financial institutions will directly affect their strategic choices.

### 3.2 Model building

#### 3.2.1 Basic assumptions and parameter settings

Hypothesis 1: This study selects government regulatory departments (*G*), banking financial institutions (*B*), non-banking financial institutions (*NB*), and high-carbon enterprises (*E*) as the leading players in the game. Compared to the traditional game theory, which assumes that all participants are entirely rational, the evolutionary game theory assumes that all four parties have limited rationality. It describes the dynamic process of each party's continuous learning, adjustment, and imitation to explain why and how different parties achieve a particular state. All parties in the game aim to maximize their interests, with government regulatory departments as the leader in promoting sustainable economic and environmental development and the ultimate goal of maximizing social benefits. Banking financial institutions, non-banking financial institutions, and high-carbon enterprises aim to maximize their interests as their primary goals.

Hypothesis 2: The government regulatory authority's strategic choice is to strictly regulate, with a strategy space of *S*_*G*_= {strict regulation, loose regulation}. The benchmark return is *r*_1_, and when the regulatory authority chooses strict regulation, it will incur regulatory costs *c*_1_. When high-carbon enterprises meet emission standards, it will improve the environmental situation, and the government will receive additional environmental benefits Δ*r*_1_. However, when enterprises exceed emission standards, the government must pay additional governance costs Δ*c*_1_. In addition, when high-carbon enterprises exceed emission standards, banks, and financial institutions do not implement green credit policies, and non-bank financial institutions do not enter the green finance market, they will be punished with *P*_4_, *P*_2_, *P*_3_, and respectively.

Hypothesis 3: The strategic choice of banking financial institutions is whether to implement green credit policies, with a strategy space of *S*_*B*_= {green credit, traditional credit}. The benchmark return of banking financial institutions is *r*_2_, and implementing green credit will incur costs *c*_2_, while also receiving government policy incentives *s*_2_. Similarly, when high-carbon enterprises strictly implement emission standards, banks will receive additional environmental benefits *e* (43, 44). The benefits mainly include the following: (1) Carbon emission compliant enterprises usually meet green credit standards, and banks can issue low-risk, high interest special loans to them, which helps reduce their overall credit risk. (2) Financial institutions investing in green bonds issued by qualified enterprises can enjoy tax incentives and secondary market premiums, further bringing them substantial investment returns. (3) Enterprises with excessive carbon emissions face risks such as fines and production shutdowns for rectification, while the environmental compliance costs of compliant enterprises have significantly decreased, which will further reduce the default probability of financial institutions and help improve their asset quality. If non-bank financial institutions also actively enter the green credit market at this time, expanding participants in the green credit market can effectively reduce information asymmetry risks and further increase the supervision of high-carbon enterprises, which will bring green premiums to banking financial institutions Δ*r*_2_. Implementing traditional credit will obtain additional risk benefits of η_2_. However, there is a loss of punishment of π_2_.

Hypothesis 4: The strategic choice of non-bank financial institutions is whether to enter the green financial market, and the strategic space is *S*_*NB*_= {enter, not enter}. The benchmark return is *r*_3_, when non-bank financial institutions choose to enter the green financial market when banking financial institutions implement green credit. Due to the existence of mature information leaders, this will greatly reduce the investment risk of non-bank financial institutions and improve the quality of their fund use, resulting in a return of Δ*r*_3_. Similarly, when banking financial institutions do not implement green credit policies, non-bank financial institutions entering the green financial market need to pay additional costs Δ*c*_3_. When enterprises actively implement emission reduction strategies, non-bank financial institutions will also receive additional environmental benefits *e* (41, 42). Similarly, when non-bank financial institutions enter the green financial market, they will receive government incentives of *s*_3_. In addition, when they do not enter the green financial market, they can obtain additional risk benefits η_3_, but they may also face fines from regulatory authorities of π_3_.

Hypothesis 5: The strategic choice for high-carbon enterprises is whether to meet emission standards, with a strategy space of *S*_*E*_= {emission standards met, emission standards exceeded}. The benchmark return for high-carbon enterprises is *r*_4_. When banks and non-bank financial institutions simultaneously enter the green credit market, high-carbon enterprises that exceed emission standards will not be able to obtain funding from banks and non-bank financial institutions, and will need to obtain funding through other channels. The additional cost to be paid at this time is *c*_41_. However, if banks and non-bank financial institutions implement green credit policies instead of banks and non-bank financial institutions not entering the green financial market, high-carbon enterprises that exceed emission standards will not be able to obtain loans from banks and non-bank financial institutions but can receive support from non-bank financial institutions. The additional cost to be paid is *c*_42_. When banks implement green credit policies, the cost of meeting emission standards for enterprises is *c*_43_. When banks do not implement green credit, the cost of meeting emission standards for enterprises is *c*_44_, and meeting emission standards will receive policy incentives from regulatory authorities (*s*_4_). The cost of exceeding emission standards can be ignored, but high-carbon enterprises will face fines from regulatory authorities (π_4_).

Hypothesis 6: Assuming that the probability of strict regulation by government regulatory authorities is *x*(0 ≤ *x* ≤ 1), the probability of choosing loose regulation is 1−*x*. The probability of banking and financial institutions choosing green credit strategies is *y*(0 ≤ *y* ≤ 1), and the probability of choosing traditional credit is 1−*y*. The probability of non-bank financial institutions choosing to enter the green finance market is *z*(0 ≤ *z* ≤ 1), and the probability of choosing not to enter is 1 −*z*. The probability of high-carbon enterprises choosing to meet emission standards is *w*(0 ≤ *w* ≤ 1), and the probability of them choosing to exceed emission standards is 1 − *w*.

#### 3.2.2 Construction of payment benefit matrix

According to the above hypothesis and parameter settings, the payment income matrix of the four parties is constructed, as shown in Table 1.

**Table 1:** Four party game payment matrix.

**Four party game subject**		**Strict supervision by government regulatory agencies (** ***x*** **)**		**Loose regulation by government regulatory agencies (**1−***x*****)**	
**Bank**	**Non-bank financial institutions**	**High carbon enterprises meet emission standards (** *w* **)**	**High carbon enterprises exceed emission standards (**1−*w***)**	**High carbon enterprises meet emission standards (** *w* **)**	**High carbon enterprises exceed emission standards (**1−*w***)**
Green Credit (*y*)	Enter (*z*)	*a*_1_ = *r*_1_−*c*_1_+Δ*r*_1_−*s*_2_−*s*_3_−*s*_4_ *b*_1_ = *r*_2_−*c*_2_+*s*_2_+Δ*r*_2_ *c*_1_ = *r*_3_+Δ*r*_3_+*s*_3_ *d*_1_ = *r*_4_−*c*_43_+*s*_4_	*a*_2_ = *r*_1_−*c*_1_−*s*_2_−*s*_3_−Δ*c*_1_+π_4_ *b*_2_ = *r*_2_−*c*_2_+*s*_2_+Δ*r*_2_ *c*_2_ = *r*_3_+Δ*r*_3_+*s*_3_ *d*_2_ = *r*_4_−*c*_41_−π_4_	*a*_3_ = *r*_1_−α*c*_1_+Δ*r*_1_ *b*_3_ = *r*_2_−*c*_2_+Δ*r*_2_ *C*_3_ = *r*_3_+Δ*r*_3_ *d*_3_ = *r*_4_−*c*_43_	*a*_4_ = *r*_1_−α*c*_1_−Δ*c*_1_ *b*_4_ = *r*_2_−*c*_2_+Δ*r*_2_ *c*_4_ = *r*_3_+Δ*r*_3_ *d*_4_ = *r*_4_−*c*_41_
	Do not enter (1−*z*)	*a*_5_ = *r*_1_−*c*_1_+Δ*r*_1_−*s*_2_−*s*_4_ *b*_5_ = *r*_2_−*c*_2_+*s*_2_ *c*_5_ = *r*_3_+η_3_ *d*_5_ = *r*_4_−*c*_43_+*S*_4_	*a*_6_ = *r*_1_−*c*_1_−*s*_2_+π_3_+π_4_−Δ*c*_1_ *b*_6_ = *r*_2_−*c*_2_+*s*_2_ *c*_6_ = *r*_3_+η_3_−π_3_ *d*_6_ = *r*_4_−*c*_42_−π_4_	*a*_7_ = *r*_1_−α*c*_1_+Δ*r*_1_ *b*_7_ = *r*_2_−*c*_2_ *c*_7_ = *r*_3_+η_3_ *d*_7_ = *r*_4_−*c*_43_	*a*_8_ = *r*_1_−α*c*_1_−Δ*c*_1_ *b*_8_ = *r*_2_−*c*_2_ *c*_8_ = *r*_3_+η_3_ *d*_8_ = *r*_4_−*c*_42_
Traditional credit (1−*y*)	Enter (*z*)	*a*_9_ = *r*_1_−*c*_1_+Δ*r*_1_−*s*_3_−*s*_4_ *b*_9_ = *r*_2_+η_2_ *c*_9_ = *r*_3_+*s*_3_−Δ*c*_3_ *d*_9_ = *r*_4_−*c*_44_+*s*_4_	*a*_10_ = *r*_1_−*c*_1_−*s*_3_+π_2_+π_4_−Δ*c*_1_ *b*_10_ = *r*_2_+η_2_−π_2_ *c*_10_ = *r*_3_+*s*_3_−Δ*c*_3_ *d*_10_ = *r*_4_−π_4_	*a*_11_ = *r*_1_−α*c*_1_+Δ*r*_1_ *b*_11_ = *r*_2_+η_2_ *c*_11_ = *r*_3_−Δ*c*_3_ *d*_11_ = *r*_4_−*c*_44_	*a*_12_ = *r*_1_−α*c*_1_−Δ*c*_1_ *b*_12_ = *r*_2_+η_2_ *c*_12_ = *r*_3_−Δ*c*_3_ *d*_12_ = *r*_4_
	Do not enter (1−*z*)	*a*_13_ = *r*_1_−*c*_1_+Δ*r*_1_−*s*_4_ *b*_13_ = *r*_2_+η_2_ *c*_13_ = *r*_3_+η_3_ *d*_13_ = *r*_4_−*c*_44_+*s*_4_	*a*_14_ = *r*_1_−*c*_1_+π_3_+π_2_+π_4_−Δ*c*_1_ *b*_14_ = *r*_2_+η_2_−π_2_ *c*_14_ = *r*_3_+η_3_−π_3_ *d*_14_ = *r*_4_−π_4_	*a*_15_ = *r*_1_−α*c*_1_+Δ*r*_1_ *b*_15_ = *r*_2_+η_2_ *c*_15_ = *r*_3_+η_3_ *d*_15_ = *r*_4_−*c*_44_	*a*_16_ = *r*_1_−α*c*_1_−Δ*c*_1_ *b*_16_ = *r*_2_+η_2_ *c*_16_ = *r*_3_+η_3_ *d*_16_ = *r*_4_

## 4 Analysis on the stability of the strategies of the game player

### 4.1 Stability analysis of strategic choices by government regulatory agencies

The expected returns of the government regulatory authorities choosing strict and loose regulatory strategies are *U*_*A*1_ and *U*_*A*2_, respectively, with an average expected return of *U*_*A*_. The replication dynamic equation and the first derivative of their behavioral strategy are shown in Equations 2, 3:


(1) $$\begin{array}{l} U_{A1}=yzwa_{1}+yz\left(1-w\right)a_{2}+y\left(1-z\right)wa_{5}+y\left(1-z\right)\left(1-w\right)a_{6}\\ \;+\left(1-y\right)zwa_{9}+\left(1-y\right)z\left(1-w\right)a_{10}+\left(1-y\right)\left(1-z\right)wa_{13}\\ \;+\left(1-y\right)\left(1-z\right)\left(1-w\right)a_{14}\\ \;U_{A2}=yzwa_{3}+yz\left(1-w\right)a_{4}+y\left(1-z\right)wa_{7}+y\left(1-z\right)\left(1-w\right)a_{8}\\ \;+\left(1-y\right)zwa_{11}+\left(1-y\right)z\left(1-w\right)a_{12}+\left(1-y\right)\left(1-z\right)wa_{15}\\ \;+\left(1-y\right)\left(1-z\right)\left(1-w\right)a_{16}\\ U_{A}=xU_{A1}+\left(1-x\right)U_{A2} \end{array}$$

(2) $$\begin{array}{l} F\left(x\right)=x\left(U_{A1}-U_{U_{A}}\right)=x\left(1-x\right)\left(U_{A1}-U_{A2}\right)\\ \;=x\left(1-x\right)f\left(y,z,w\right) \end{array}$$

Among them:


(3) $$\begin{array}{l} f(y,z,w)=-[c_{1}-\pi_{2}-\pi_{3}-\pi_{4}+(s_{4}+\pi_{2}+\pi_{3}+\pi_{4})w\\ \;+(s_{2}+\pi_{2})y+(s_{3}+\pi_{3})z-\pi_{2}wy-\pi_{3}wz]\\ \;F^{\prime }(x)=(1-2x)f(y,z,w) \end{array}$$

According to the stability theorem of differential equations, the strategic choice of government regulatory authorities in a stable state needs to satisfy: *F*(*x*) = 0 and *F*′(*x*) < 0.

**Proposition 1:**

- When *w* < *w*_0_, the stable strategy choice of the government regulatory department is “strict supervision.”
- When *w* > *w*_0_, the stable strategy choice of the government regulatory department is “relaxed supervision.”
- When *w* = *w*_0_, the stable strategy choice of the government regulatory department cannot be determined. The threshold value is:

$$\begin{array}{l} w_{0}=-[c_{1}-\pi_{2}-\pi_{3}-\pi_{4}+(s_{2}+\pi_{2})y+(s_{3}+\pi_{3})z]/(s_{4}\\ \;+\pi_{2}+\pi_{3}+\pi_{4}-\pi_{2}y-\pi_{3}z). \end{array}$$

**Proof:** Since ∂*f*(*y, z, w*)/∂*w* < 0, it follows that is a decreasing function of *f*(*y, z, w*). When *w*<*w*_0_, we have *f*(*y, z, w*)>0, *F*(*x*)|_*x* = 1_ = 0 and $F^{\prime }\left(x\right){|}_{x=1}<0$, which implies *x* = 1 stability. When *w*>*w*_0_, we have *f*(*y, z, w*) < 0, *F*(*x*)|_*x* = 0_ = 0 and $F^{\prime }\left(x\right){|}_{x=0}<0$, which *x* = 0 implies stability. When *w* = *w*_0_, we have *F*(*x*) = 0 and *F*′(*x*) = 0, which makes it impossible to determine a stable strategy, and it is proved.

According to Proposition 1, it can be concluded that in the strategic choice of government regulatory departments, if the probability of high-carbon enterprises meeting emission standards is low, the strategic choice of government regulatory departments will be “strict supervision.” As the environmental awareness of high-carbon enterprises gradually increases, and the willingness to meet emission standards gradually increases and exceeds a certain threshold, the regulatory departments will reduce the intensity of supervision and progressively shift to “relaxed supervision.”

**Figure 2:**
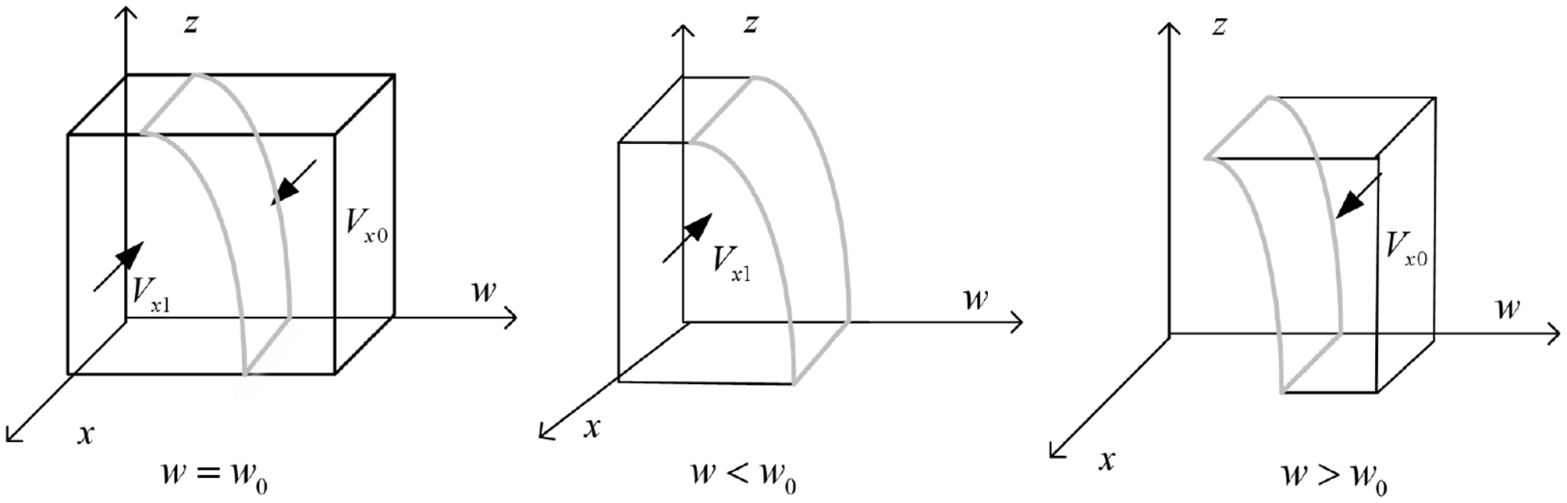
Phase diagram of government regulatory department strategy selection.

The phase diagram of government strategy selection is drawn according to Proposition 1 is shown in Figure 2.

As shown in Figure 2, the volume of part *V*_*x*1_ is the probability of the government regulatory department choosing “strict supervision,” and the volume of part *V*_*x*0_ is the probability of the government regulatory department choosing “loose supervision.” It is calculated that:


$$\begin{array}{l} t_{1}=-\left[c_{1}-\pi_{2}-\pi_{3}-\pi_{4}+\left(s_{2}+\pi_{2}\right)y\right],\\ t_{2}=\left(s_{4}+\pi_{2}+\pi_{3}+\pi_{4}-\pi_{2}y\right) \end{array}$$

The calculation shows that


(4) $$\begin{array}{l} V_{x1}\text{=}\int_{0}^{1}\int_{0}^{1}\left[t_{1}-(s_{3}+\pi_{3})z\right]/(t_{2}-\pi_{3}z)dzdx=[-\pi_{3}t_{1}\\ \;+(s_{3}+\pi_{3})t_{2}]\ln(1-\pi_{3}/t_{2})/[\pi_{3}^{2}-(s_{3}+\pi_{3})/\pi_{3}] \end{array}$$

Then there are:


(5) $$\begin{array}{l} V_{x0}=1-V_{x1}=1-[-\pi_{3}t_{1}+(s_{3}+\pi_{3})t_{2}]\ln(1-\pi_{3}/t_{2})/[{\pi_{3}}^{2}\\ \;-(s_{3}+\pi_{3})/\pi_{3}] \end{array}$$

**Inference 1.1:** The strategic choice of government regulatory departments is mainly influenced by their regulatory costs and the implementation of reward and punishment mechanisms. That is, when the regulatory expenses of government regulatory departments are too high, even if they may face high subsequent additional governance costs, the probability of strict regulation by the government is relatively low. Similarly, when the government regulatory departments provide more lavish subsidies to the other three parties and gradually increase their punishment, they will be more inclined to develop in a direction that benefits the social environment and economic benefits. At this time, the government does not need to pay too much attention to the green financial market and the carbon emissions of enterprises, so the probability of choosing strict regulation will gradually decrease.

**Proof:** Calculate the first derivative of *V*_*x*1_ for π_2_, π_3_, π_4_, *s*_2_, *s*_3_, *s*_4_, and *c*_1_ to obtain: ∂*V*_*x*1_/∂*c*_1_ < 0, ∂*V*_*x*1_/∂π_2_>0, ∂*V*_*x*1_/∂π_3_>0, ∂*V*_*x*1_/∂π_4_>0, ∂*V*_*x*1_/∂*s*_2_ < 0, ∂*V*_*x*1_/∂*s*_3_ < 0, and ∂*V*_*x*1_/∂*s*_4_ < 0. It can be seen that *V*_*x*1_ is negatively correlated with *s*_2_, *s*_3_, *s*_4_, and *c*_1_, and positively correlated with π_2_, π_3_, and π_4_. According to the aforementioned, let *f*(*c*_1_) = −[*c*_1_−π_2_−π_3_−π_4_+(*s*_4_+π_2_+π_3_+π_4_)*w*+(*s*_2_+π_2_)*y*+(*s*_3_+π_3_)*z*−π_2_*wy*−π_3_*wz*], then ∂*f*/∂*c*_1_ < 0, we can see that *f* is a decreasing function with respect to *C*_1_, where:


$$\begin{array}{l} c_{0}=-[-\pi_{2}-\pi_{3}-\pi_{4}+(s_{4}+\pi_{2}+\pi_{3}+\pi_{4})w+(s_{2}+\pi_{2})y\\ \;+(s_{3}+\pi_{3})z-\pi_{2}wy-\pi_{3}wz] \end{array}$$

When *c*_1_ > *c*_0_, there are *F*(*x*)|_*x* = 0_ = 0 and $F^{\prime }\left(x\right){|}_{x=0}\;<\;0$. Currently, the government regulatory authorities' strategic choices tended to be “relaxed supervision.” When *c*_1_<*c*_0_, there are *F*(*x*)|_*x* = 1_ = 0 and $F^{\prime }\left(x\right){|}_{x=1}<0$. The government regulatory authorities' strategic choices tend to be “strict supervision.” Therefore, the government regulatory authorities' strategic decisions are mainly influenced by their regulatory costs. When the regulatory costs are low, the authorities are more inclined to adopt strict supervision strategies. However, when the regulatory costs gradually increase and exceed a certain threshold, the government regulatory authorities will choose “relaxed supervision,” even if they face higher environmental governance costs in the future.

### 4.2 Analysis of the strategic stability of banking financial institutions

The expected values of the banking financial institutions choosing the “green credit” and “traditional credit” strategies are *U*_*B*1_ and *U*_*B*2_, respectively, with an average return of *U*_*B*_. The replication dynamic equation and the first derivative of their behavioral strategy are shown in Equations 7, 8:


(6) $$\begin{array}{l} U_{B1}=xzwb_{1}+xz(1-w)b_{2}+x(1-z)wb_{5}+x(1-z)(1\\ \;-w)b_{6}+(1-x)zwb_{3}+(1-x)z(1-w)b_{4}+(1\\ \;-x)(1-z)wb_{7}+(1-x)(1-z)(1-w)b_{8}\\ U_{B2}=xzwb_{9}+xz(1-w)b_{10}+x(1-z)wb_{13}+x(1-z)(1\\ \;-w)b_{14}+(1-x)zwb_{11}+(1-x)z(1-w)b_{12}+(1\\ \;-x)(1-z)wb_{15}+(1-x)(1-z)(1-w)b_{16}\\ U_{B}=yU_{B1}+\left(1-y\right)U_{B2} \end{array}$$

(7) $$\begin{array}{l} F\left(y\right)=y\left(U_{B1}-U_{B}\right)=y\left(1-y\right)\left(U_{B1}-U_{B2}\right)\\ \;=y\left(1-y\right)f_{1}\left(x,z,w\right) \end{array}$$

(8) $$\begin{array}{l} f_{1}\left(x,z,w\right)=z\Delta r_{2}-\eta_{2}-c_{2}+\left(s_{2}+\pi_{2}\right)x-\pi_{2}wx\\ \;F^{\prime }\left(y\right)=\left(1-2y\right)f_{1}\left(x,z,w\right) \end{array}$$

According to the stability theorem of differential equations, the strategy of banking financial institutions in a stable state should meet the following requirements: *F*(*y*) = 0 and *F*′(*y*) < 0

**Proposition 2**

- When *w* < *w*_1_, the strategic choice of banking financial institutions tends to be green credit strategy.
- When *w* > *w*_1_, the strategic choice of banking financial institutions tends to be traditional credit strategy.
- When *w* = *w*_0_, the stable strategy of banking financial institutions cannot be determined. The threshold values are:

$$\begin{array}{l} w_{1}=\left[z\Delta r_{2}-\eta_{2}-c_{2}+\left(s_{2}+\pi_{2}\right)x\right]/\pi_{2}x \end{array}$$

**Proof:** It can be seen from ∂*f*_1_(*x, z, w*)/∂*w* < 0 that *f*_1_(*x, z, w*) is a decreasing function of *w*. When *w*<*w*_1_, there are *f*_1_(*x, z, w*)>0, *F*(*y*)|_*y* = 1_ = 0 and $F^{\prime }\left(y\right){|}_{y=1}<0$, where *y* = 1 is stable; When *w*>*w*_1_, there are *f*_1_(*x, z, w*) < 0, *F*(*y*)|_*y* = 0_ = 0 and $F^{\prime }\left(y\right){|}_{y=0}<0$, where *y* = 0 is stable; When *w* = *w*_1_, *F*(*y*) = 0 and *F*(*y*′) = 0, and the stability cannot be determined at this time, which is verified.

According to Proposition 2, the strategic choices of banking financial institutions are closely related to the strategic decisions of high-carbon enterprises. When the probability of high-carbon enterprises meeting emission standards is relatively low, the strategic choice of banking financial institutions is to provide “green credit” to assist enterprises in green transformation. However, with the changes in high-carbon enterprises concept and environmental awareness have enhanced, the strategic choice will gradually shift to meeting emission standards. This means that the implementation of green credit policies by banking and financial institutions has achieved good results. The implementation intensity will gradually decrease, ultimately still dominated by traditional credit policies.

**Figure 3:**
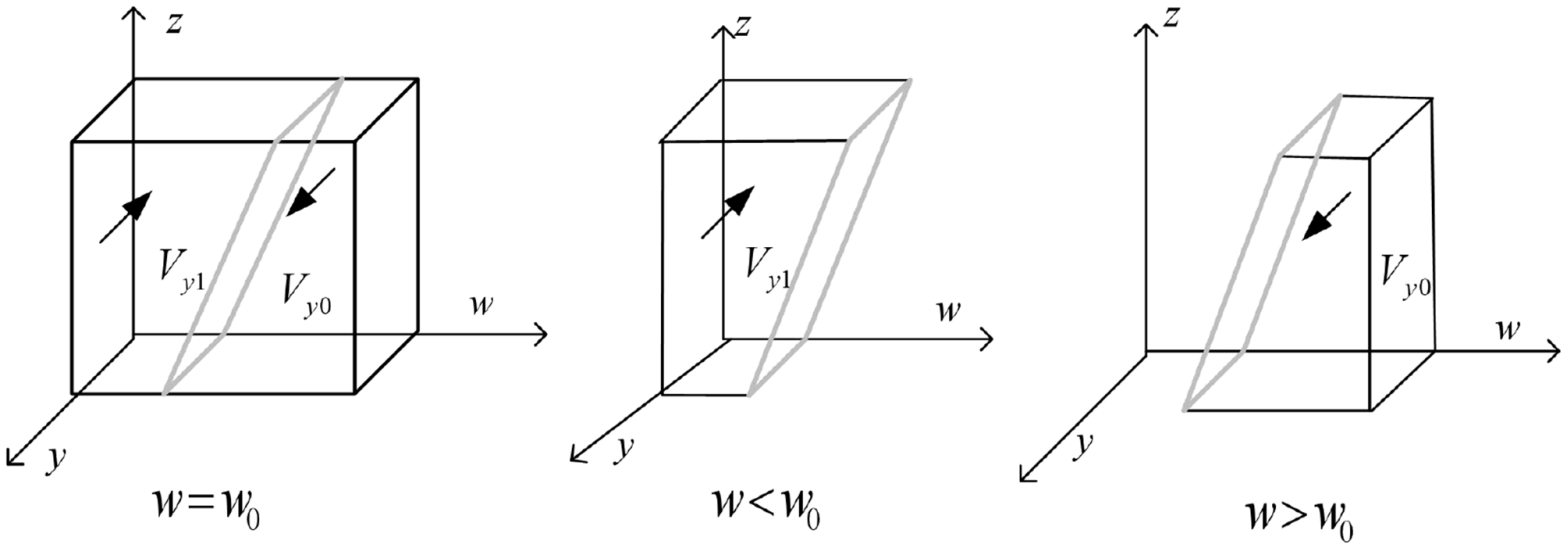
Phase diagram of strategy selection for banks and financial institutions.

Draw the phase diagram of strategy selection of bank financial institutions according to Proposition 2, as shown in Figure 3:

As shown in Figure 3, the volumes of the *V*_*y*0_ and *V*_*y*1_ parts represent the probabilities of bank financial institutions choosing traditional credit and green credit strategies, respectively, and it is calculated that:


(9) $$\begin{array}{l} V_{y1}=\int_{0}^{1}\int_{0}^{1}\left[z\Delta r_{2}-\eta_{2}-c_{2}+\left(s_{2}+\pi_{2}\right)x\right]/\left(\pi_{2}x\right)dzdy\\ \;=\left[\Delta r_{2}/2-\eta_{2}-c_{2}+\left(s_{2}+\pi_{2}\right)x\right]/\pi_{2}x \end{array}$$

Then there are:


(10) $$\begin{array}{l} V_{y0}=1-\left[\Delta r_{2}/2-\eta_{2}-c_{2}+\left(s_{2}+\pi_{2}\right)x\right]/\pi_{2}x \end{array}$$

**Inference 2.1:** The choice of green credit strategies for banking financial institutions is mainly influenced by the costs and benefits of policy implementation, with a positive correlation with the benefits of green credit and a negative correlation with the costs and benefits of traditional credit. In addition, the subsidies and penalties imposed by government regulatory agencies also impact the choice of green credit strategies for banking financial institutions.

**Proof:** Calculate the first derivative of the probability *V*_*y*1_ of a bank financial institution choosing a green credit strategy for Δ*r*_2_, *c*_2_, η_2_, *s*_2_, π_2_ to obtain: ∂*V*_*y*1_/∂△*r*_2_>0, ∂*V*_*y*1_/∂*s*_2_>0, ∂*V*_*y*1_/∂π_2_>0, ∂*V*_*y*1_/∂η_2_ < 0, ∂*V*_*y*1_/∂*c*_2_ < 0. From this, it can be seen that *V*_*y*1_ is positively correlated with Δ*r*_2_, *s*_2_, π_2_, and negatively correlated with η_2_, *c*_2_. The certificate is completed.

**Inference 2.2:** There is a certain threshold for government regulatory authorities to subsidize banking institutions in implementing green credit policies and impose penalties for non-implementation of green credit. When the subsidy is small, banking institutions consider their benefits and may face more significant uncertainty risks, and their strategic choices tend to be traditional credit. When the subsidy value exceeds a certain threshold, the strategic decisions of banking institutions will gradually shift toward actively implementing green credit strategies. Similarly, there is a threshold for the penalties imposed on banking institutions for not implementing green credit strategies. Below the threshold, the penalties are relatively small, and banking institutions are more inclined to traditional credit. Considering the vast penalty costs, banking institutions will gradually shift toward implementing green credit rather than traditional credit when the threshold is exceeded.

**Proof:** Let *f*_1_(*s*_2_) = *zΔr*_2_−η_2_−*c*_2_+(*s*_2_+π_2_)*x*−π_2_*wx*, then ∂*f*_1_(*s*_2_)/∂*s*_2_>0, we can see that *f*_1_ is an increasing function of *s*_2_, where $s_{2}^{*}=\left(z\Delta r_{2}-\eta_{2}-c_{2}+\pi_{2}x-\pi_{2}wx\right)/x$. When $s_{2}>s_{2}^{*}$, there are *F*(*y*)|_*y* = 1_ = 0 and $F^{\prime }\left(y\right){|}_{y=1}<0$, and the strategy choice of banking financial institutions tends to implement green credit. Similarly, when $s_{2}<s_{2}^{*}$, there are *F*(*y*)|_*y* = 0_ = 0 and $F^{\prime }\left(y\right){|}_{y=0}<0$, and the strategy choice of banking financial institutions tends to traditional credit. And the same can be proved π_2_. Therefore, before the penalty for choosing traditional credit strategies reaches a threshold, banks will not be prompted to implement green credit strategies. Still, when the penalty value exceeds a certain threshold, banking financial institutions will most likely choose to implement green credit policies.

### 4.3 Stability analysis of non-bank financial institutions' strategy selection

The expected returns for non-bank financial institutions to choose to enter and not enter the green finance market are *U*_*C*1_ and *U*_*C*2_, respectively, with an average expected return of *U*_*C*_. The replication dynamic equation and the first derivative of their behavior strategy are shown in Equations 12, 13:


(11) $$\begin{array}{l} U_{C1}=xywc_{1}+xy(1-w)c_{2}+(1-x)ywc_{3}+(1-x)y(1\\ \;-w)c_{4}+x(1-y)wc_{9}+x(1-y)(1-w)c_{10}\\ \;+(1-x)(1-y)wc_{11}+(1-x)(1-y)(1-w)c_{12}\\ U_{C2}=xywc_{5}+xy(1-w)c_{6}+(1-x)ywc_{7}+(1-x)y(1\\ \;-w)c_{8}+x(1-y)wc_{13}+x(1-y)(1-w)c_{14}+(1\\ \;-x)(1-y)wc_{15}+(1-x)(1-y)(1-w)c_{16}\\ U_{C}=zU_{C1}+\left(1-z\right)U_{C2} \end{array}$$

(12) $$\begin{array}{l} F\left(z\right)=z\left(U_{C1}-U_{C}\right)=z\left(1-z\right)\left(U_{C1}-U_{C2}\right)\\ \;=z\left(1-z\right)f_{2}\left(x,y,w\right) \end{array}$$

(13) $$\begin{array}{l} f_{2}\left(x,y,w\right)=\left(\Delta c_{3}+\Delta r_{3}\right)y-\eta_{3}-\Delta c_{3}+\left(s_{3}+\pi_{3}\right)x-\pi_{3}wx\\ \;F^{\prime }\left(z\right)=\left(1-2z\right)f_{2}\left(x,y,w\right) \end{array}$$

According to the stability theorem of differential equations, the strategy of non-bank financial institutions in a stable state shall meet the following requirements: *F*(*z*) = 0 and *F*′(*z*) < 0.

**Proposition 3:**

- When *y* < *y*_1_, the strategy choice of non-bank financial institutions is not to enter the green financial market.
- When *y* < *y*_1_, the strategy choice of non-bank financial institutions is to enter the green financial market.
- When *y* = *y*_1_, it is impossible to determine the stable strategy of non-bank financial institutions, where the threshold value is: *y*_0_ = −[−η_3_−Δ*c*_3_+(*s*_3_+π_3_)*x*−π_3_*wx*]/(Δ*c*_3_+Δ* r*_3_)

**Proof:** ∂*f*_2_(*x, y, w*)/∂*y*>0, *f*_1_(*x, y, w*) is an increasing function of *y*, when *y*<*y*_0_, there are *f*_2_(*x, y, w*) < 0, *F*(*z*)|_*z* = 0_ = 0 and $F^{\prime }\left(z\right){|}_{z=0}<0$, and *z* = 0 is stable; when *y*>*y*_0_, there are *f*_2_(*x, y, w*) < 0, *F*(*z*)|_*z* = 1_ = 0 and $F^{\prime }\left(z\right){|}_{z=1}<0$, and *z* = 1 is stable; when *y* = *y*_0_, *F*(*z*) = 0 and *F*′(*z*) = 0, which cannot determine the stable strategy of non-bank financial institutions. Proof completed.

According to Proposition 3, with the gradual deepening of green concepts and the gradual expansion of the scale of green financial markets, banking financial institutions with information advantages will transmit green development concepts to non-bank financial institutions in the implementation of green credit strategies, which means that signal effects exist in the financial system, which will further affect the decision-making of non-bank financial institutions to enter the green financial market. When banking financial institutions gradually increase their willingness to implement green credit policies, non-bank financial institutions are more likely to follow and join the green financial market due to the influence of signal transmission effects. At this time, both parties learn, imitate, and compete with each other, which can effectively compensate for the lack of supply of green credit funds in the market and is also of great significance for the innovative development of the green financial market.

**Figure 4:**
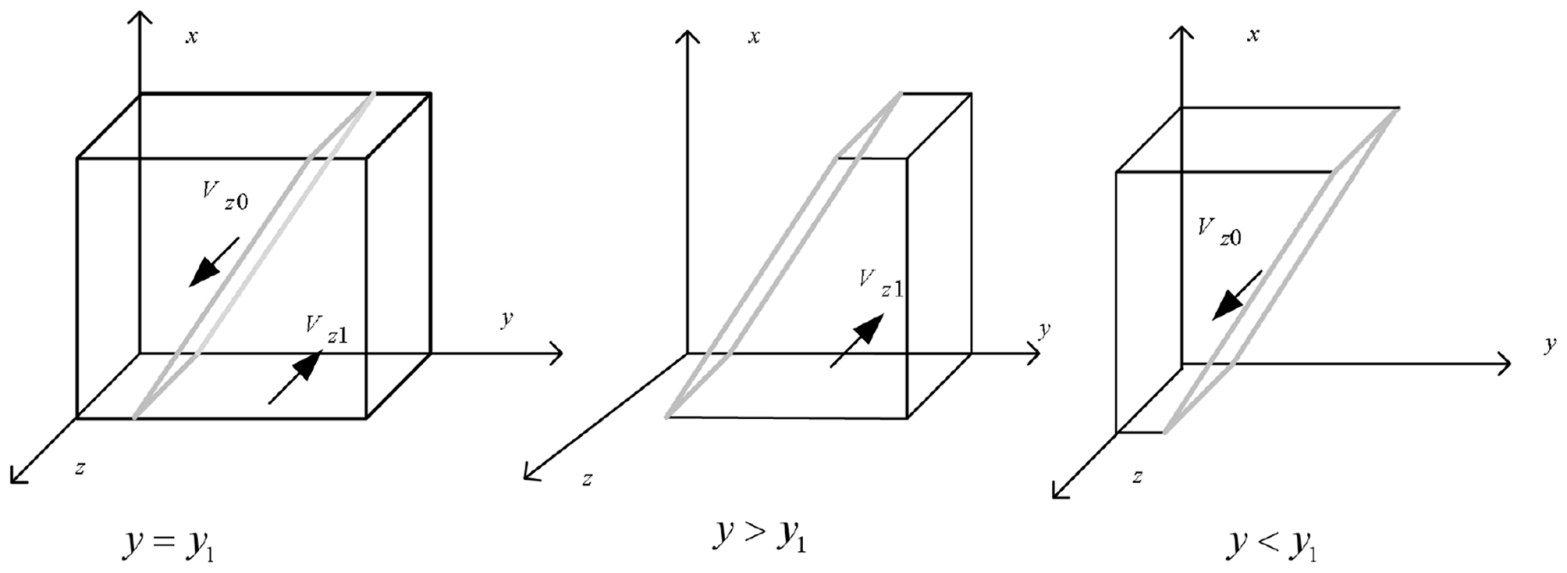
Phase diagram of strategy selection for non-bank financial institutions.

Draw the phase diagram of non-bank financial institutions' strategic choices according Proposition 3, as shown in Figure 4.

It can be seen from Figure 4 that the volume of part *V*_*z*0_ and part *V*_*z*1_ is the non-bank financial institutions choosing not to enter and enter the green financial market is respectively. And it is calculated that:


(14) $$\begin{array}{l} V_{z0}=\int_{0}^{1}\int_{0}^{1}-[-\eta_{3}-\Delta c_{3}+(s_{3}+\pi_{3})x-\pi_{3}wx]/(\Delta c_{3}\\ \;+\Delta r_{3})dxdz\\ \;=-[-\eta_{3}-\Delta c_{3}+(s_{3}+\pi_{3})/2-\pi_{3}w/2]/(\Delta c_{3}+\Delta r_{3}) \end{array}$$

(15) $$\begin{array}{l} V_{z1}=1-V_{z0}=1+[-\eta_{3}-\Delta c_{3}+(s_{3}+\pi_{3})/2-\pi_{3}w/2]/(\Delta c_{3}\\ \;+\Delta r_{3}) \end{array}$$

**Inference 3.1**: The strategic choice of non-bank financial institutions is mainly influenced by the costs and benefits of entering the green financial market. The increase in the benefits and decrease in the costs of non-bank financial institutions entering the green financial market will increase the probability of non-bank financial institutions entering the green financial market. However, when the benefits of not entering the green financial market increase and the costs and penalties decrease, the probability of non-bank financial institutions entering the green financial market will decrease. Therefore, to encourage non-bank financial institutions to enter the green financial market actively, government regulatory authorities should improve the implementation of subsidy mechanisms. In addition, bank financial institutions should also be encouraged to fully utilize their information advantages and actively convey the concept of green development in implementing green credit policies to attract more entities to enter the green financial market and provide strong support for high-quality green development of the economy.

**Proof:** The first partial derivative of the probability of the bank implementing the green credit policy ***V***_***z*****1**_ vs. π_3_, η_3_, Δ*c*_3_, and *s*_3_ is obtained: ∂*V*_*z*1_/∂η_3_ < 0, ∂*V*_*z*1_/∂*Δc*_3_ < 0, ∂*V*_*z*1_/∂π_3_>0 and ∂*V*_*z*1_/∂*s*_3_>0. Proof completed.

**Inference 3.2:** There is a threshold for government regulatory authorities to implement the reward and punishment mechanism for non-bank financial institutions. When below the threshold, non-bank financial institutions tend to choose not to enter the green financial market; when the punishment and incentive are both above the threshold, the strategic choice of non-bank financial institutions will gradually shift from not entering to entering the green financial market.

**Proof:** Assuming *f*_2_(*s*_3_) = (Δ*c*_3_+Δ*r*_3_)*y*−η_3_−Δ*c*_3_+(*s*_3_+π_3_)*x*−π_3_*wx*, we have ∂*f*_2_(*s*_3_)/∂*s*_3_>0, We can get *f*_2_(*s*_3_) is an increasing function of *s*_3_, where:


$$\begin{array}{l} s_{3}^{*}=\left[\left(\Delta c_{3}+\Delta r_{3}\right)y-\eta_{3}-\Delta c_{3}+\pi_{3}x-\pi_{3}wx\right]/x \end{array}$$

When $s_{3}<{s_{3}}^{*}$, there are *F*(*z*)|_*z* = 0_ = 0 and $F^{\prime }\left(z\right){|}_{z=0}<0$, where *z* = 0 is stable; when $s_{3}>s_{3}^{*}$, there are *F*(*z*)|_*z* = 1_ = 0 and $F^{\prime }\left(z\right){|}_{z=1}<0$, where *z* = 1 is stable, proof completed.

### 4.4 Stability of high-carbon enterprise strategy selection

The expected benefits of high-carbon enterprises choosing strategies to meet and exceed emission standards are *U*_*D*1_ and *U*_*D*2_, respectively, with an average anticipated benefit of *U*_*D*_. he replication dynamic equation and the first derivative of their behavioral strategies are shown in Equations 17, 18:


(16) $$\begin{array}{l} U_{D1}=xyzd_{1}+xy(1-z)d_{5}+(1-x)yzd_{3}+(1-x)y(1\\ \;-z)d_{7}+x(1-y)zd_{9}+x(1-y)(1-z)d_{13}+(1-x)(1\\ \;-y)zd_{11}+(1-x)(1-y)(1-z)d_{15}\\ U_{D2}=xyzd_{2}+xy(1-z)d_{6}+(1-x)yzd_{4}+(1-x)y(1\\ \;-z)d_{8}+x(1-y)zd_{10}+x(1-y)(1-z)d_{14}\\ \;+(1-x)(1-y)zd_{12}+(1-x)(1-y)(1-z)d_{16}\\ U_{D}=wU_{D1}+\left(1-w\right)U_{D2} \end{array}$$

(17) $$\begin{array}{l} F\left(w\right)=w\left(U_{D1}-U_{D}\right)=w\left(1-w\right)\left(U_{D1}-U_{D2}\right)=f_{3}\left(x,y,z\right) \end{array}$$

(18) $$\begin{array}{l} f_{3}\left(x,y,z\right)=\left(c_{42}-c_{43}+c_{44}\right)y-c_{44}+\left(s_{4}+\pi_{4}\right)x+\left(c_{41}-c_{42}\right)yz\\ \;F^{\prime }\left(w\right)=\left(1-2w\right)f_{3}\left(x,y,z\right) \end{array}$$

According to the stability theorem of differential equations, the strategy of high-carbon enterprises in a stable state needs to meet the following conditions: *F*(*w*) = 0 and *F*′(*w*) < 0.

**Proposition 4**

- When *x* > *x*_0_, the stable strategy of high-carbon enterprises is to meet emission standards.
- When *x* < *x*_0_, the stable strategy of high-carbon enterprises is to exceed emission standards.
- When *x* = *x*_0_, it is impossible to determine the stable strategy of high-carbon enterprises, with the threshold value being:

$$\begin{array}{l} x_{0}=-\left[\left(c_{42}-c_{43}+c_{44}\right)y-c_{44}+\left(c_{41}-c_{42}\right)yz\right]/\left(s_{4}+\pi_{4}\right) \end{array}$$

**Proof:** Since ∂*f*_3_(*x, y, z*)/∂*x*>0, then *f*_3_(*x, y, z*) is an increasing function of *x*. When *x*>*x*_0_, there are *f*_3_(*x, y, z*)>0, *F*(*w*)|_*w* = 1_ = 0, and $F^{\prime }\left(w\right){|}_{w=1}<0$, then *w* = 1 is stable. High-carbon enterprises will choose emission compliance strategies; When *x*<*x*_0_, there are *f*_3_(*x, y, z*) < 0, *F*(*w*)|_*w* = 0_ = 0, $F^{\prime }\left(w\right){|}_{w=0}<0$, then *w* = 0 is stable and high-carbon enterprises will choose the emission excess strategy. Similarly, it can be proved that the above conditions are also met *y* and *z*, proof completed.

According to Proposition 4, for high-carbon enterprises, green transformation will face higher transformation costs, which will bring more significant financial pressure to the enterprise. Currently, the regulatory authority's supervision strength is whether banks and non-bank financial institutions can provide sufficient green funds to assist the enterprise in green transformation and achieve emission standards to promote high-quality economic development. When the regulatory authority tends to be loose in supervision, and the probability of banks and non-bank financial institutions entering the green financial market is low, high-carbon enterprises are likelier to choose to exceed emission standards. However, as the regulatory authority strengthens its supervision, banks and non-bank financial institutions implement green credit policies, and high-carbon enterprises will gradually shift to achieve emission standards.

**Figure 5:**
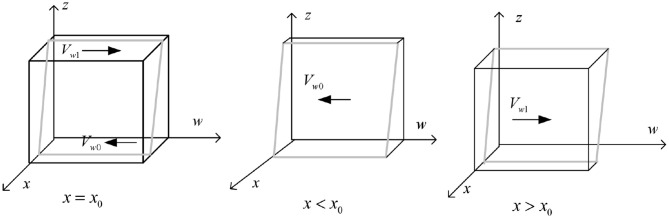
Phase diagram of strategy selection for high-carbon enterprises.

Draw the phase diagram of high-carbon enterprise strategy selection according to above Proposition, as shown in Figure 5.

It can be seen from Figure 5 that the volume of part *V*_*w*0_ and part *V*_*w*1_, represent the probabilities of high-carbon enterprises choosing strategies for exceeding emission standards and meeting emission standards, respectively. Which is calculated as follows:


(19) $$\begin{array}{l} V_{w0}=\int_{0}^{1}\int_{0}^{1}\left[-[(c_{42}-c_{43}+c_{44})y-c_{44}+(c_{41}-c_{42})yz\right]/(s_{4}\\ \;+\pi_{4})]dzdw\\ \;=-[(c_{42}-c_{43}+c_{44})y-c_{44}+(c_{41}-c_{42})y/2]/(s_{4}+\pi_{4}) \end{array}$$

Then there are:


(20) $$\begin{array}{l} V_{w1}=1-V_{w0}=1+[(c_{42}-c_{43}+c_{44})y-c_{44}+(c_{41}\\ \;-c_{42})y/2]/(s_{4}+\pi_{4}) \end{array}$$

**Inference 4.1:** The strategic choices of high-carbon enterprises are mainly influenced by their own cost-benefit and external reward and punishment mechanisms. When the benefits of meeting emission standards, the costs of exceeding emission standards, and the punishment imposed by other third-party game players on enterprises for exceeding emission standards increase, the probability of high-carbon enterprises choosing to meet emission standards will increase. However, when the benefits of meeting emission standards and the costs of exceeding emission standards increase, the probability of high-carbon enterprises choosing to meet emission standards will decrease.

**Proof:** By calculating the first partial derivatives of the probability *V*_*w*1_ of high-carbon enterprises meeting emission standards for *c*_41_, *c*_42_, *c*_43_, *c*_44_, *s*_4_, and π_4_. We can get ∂*V*_*w*1_/∂*c*_41_>0, ∂*V*_*w*1_/∂*c*_42_>0, ∂*V*_*w*1_/∂*c*_43_ < 0, ∂*V*_*w*1_/∂*c*_44_ < 0, ∂*V*_*w*1_/∂*s*_4_>0 and ∂*V*_*W*1_/∂π_4_>0, proof completed.

**Inference 4.2:** There is a threshold for the government regulatory authorities' incentives and penalties for high-carbon enterprises. When the incentives and penalties are relatively small, the strategic choices of high-carbon enterprises tend to exceed the emission standards. However, when the incentives and penalties gradually increase and exceed a certain threshold, the strategic decisions of high-carbon enterprises will shift progressively toward meeting the emission standards. Similarly, there is a threshold for punishing high-carbon enterprises' strategic choices after implementing green credit policies by banks and non-bank institutions.

**Proof:** if *f*_3_(π_4_) = (*c*_42_−*c*_43_+*c*_44_)*y*−*c*_44_+(*s*_4_+π_4_)*x*+(*c*_41_−*c*_42_)*yz*, we can calculate ∂*f*_3_(*P*_4_)/∂π_4_>0 and know that *f*_3_(π_4_) is an increasing function of π_4_. Therefore, when $\pi_{4}<{\pi_{4}}^{*}$, there are *F*(*w*)|_*w* = 1_ = 0 and $F^{\prime }\left(w\right){|}_{w=1}<0$, and *w* = 0 is stable. At this time, high-carbon enterprises tend to choose the strategy of exceeding emission standards; When $\pi_{4}>{\pi_{4}}^{*}$, there are *F*(*w*)|_*w* = 1_ = 0 and $F^{\prime }\left(w\right){|}_{w=1}<0$, and *w* = 1 is stable, At this time, high-carbon enterprises tend to choose the strategy of meeting emission standards, where ${\pi_{4}}^{*}=-\left[\left(c_{42}-c_{43}+c_{44}\right)y-c_{44}+s_{4}x+\left(c_{41}-c_{42}\right)yz\right]/x$, Similarly, it can be proven that *s*_4_. The certificate is completed.

## 5 Stability analysis of strategy combination

### 5.1 An analysis of the equilibrium point of the four-way evolutionary game of government regulatory departments, bank financial institutions, non-bank financial institutions, and high-carbon enterprises

Under the background of high-quality development, to explore the conditions and formation process of strategy selection of different participating subjects in the process of green finance helping high-carbon enterprises to reduce carbon emissions in the construction of a dynamic replication system of the four-party game of government regulatory departments, bank financial institutions, non-bank financial institutions, and high carbon enterprises, the stability of the strategy group of the four-party entities can be judged Lyapunov's first rule. Ritzberger and Weibull (45) and Selten (46) pointed out that in the multi-group evolutionary game, the stable solution of the evolutionary game is the strict Nash equilibrium, and the strict Nash equilibrium is a pure strategy. Therefore, this paper only analyzes the stability of16 groups of pure strategy equilibrium solutions and constructs the Jacobian matrix of the four-party agent replication dynamic system as follows:


(21) $$\begin{array}{l} J=\left[\begin{array}{c} \partial F\left(x\right)/\partial x\;\partial F\left(x\right)/\partial y\;\partial F\left(x\right)/\partial z\;\partial F\left(x\right)/\partial w\\ \partial F\left(y\right)/\partial x\;\partial F\left(y\right)/\partial y\;\partial F\left(y\right)/\partial z\;\partial F\left(y\right)/\partial w\\ \partial F\left(z\right)/\partial x\;\partial F\left(z\right)/\partial y\;\partial F\left(z\right)/\partial z\;\partial F\left(z\right)/\partial w\\ \partial F\left(w\right)/\partial x\;\partial F\left(w\right)/\partial y\;\partial F\left(w\right)/\partial z\;\partial F\left(w\right)/\partial w \end{array}\right] \end{array}$$

### 5.2 Result discussion stability condition analysis

According to Lyapunov's first rule, if the sign of the eigenvalues of the Jacobian matrix is negative, then the equilibrium point is asymptotically stable; If at least one of the eigenvalues of the Jacobian matrix is positive, then this point is unstable. Table 2 shows that equilibrium points *E*_2 − 5_, *E*_7 − 8_, *E*_12 − 13_, and *E*_16_ are unstable points, which will not be discussed too much here. The stability analysis of the rest of the equilibrium points is as follows:

**Table 2:** Gradual stability analysis of the equilibrium point of the dynamic system of four-party agent replication.

**Equilibrium point**	**Characteristic value ψ_1_, ψ_2_, ψ_3_, ψ_4_**	**Symbol**	**Stability and conditions**
*E*_1_ (0,0,0,0)	π_2_+π_3_+π_4_−*c*_1_, −η_2_−*c*_2_, −η_3_−Δ*c*_3_, −*c*_44_	×−−−	Stable when conditions 1 are met
*E*_2_ (0,0,0,1)	−(*c*_1_+*s*_4_), −η_2_−*c*_2_, −η_3_−Δ*c*_3_, *c*_44_	−−−+	Instability
*E*_3_ (0,0,1,0)	−(*c*_1_−π_2_−π_4_+*s*_3_), Δ*r*_2_−η_2_−*c*_2_, η_3_+Δ*c*_3_, −*c*_44_	× × +−	Instability
*E*_4_ (0,0,1,1)	*c*_1_+*s*_4_−π_3_, Δ*r*_2_−η_2_−*c*_2_, η_3_+Δ*c*_3_, *c*_44_	× × ++	Instability
*E*_5_ (0,1,0,0)	−(*c*_1_−π_3_−π_4_+*s*_2_), η_2_+*c*_2_, Δ*r*_3_−η_3_, *c*_42_−*c*_43_	×+ × −	Instability
*E*_6_ (1,0,0,0)	*c*_1_−π_2_−π_3_−π_4_, −η_2_−*c*_2_+*s*_2_+π_2_, −η_3_−Δ*c*_3_+*s*_3_+π_3_, −*c*_44_+*s*_4_+π_4_	× × × ×	Stable when conditions 2-5 are met
*E*_7_ (1,0,0,1)	*c*_1_+*s*_4_, Δ*r*_2_−η_2_−*c*_2_+*s*_2_+π_2_, −η_3_−Δ*c*_3_+*s*_3_, −*c*_44_+*s*_4_+π_4_	+ × × ×	Instability
*E*_8_ (0,1,0,1)	−(*c*_1_+*s*_2_+*s*_4_), η_2_+*c*_2_, Δ*r*_3_−η_3_, −(*c*_42_−*c*_43_)	−+ × ×	Instability
*E*_9_ (0,1,1,0)	−(*c*_1_−π_4_+*s*_2_+*s*_3_), −(Δ*r*_2_−η_2_−*c*_2_), −(Δ*r*_3_−η_3_), *c*_41_−*c*_43_	× × × ×	Stable when conditions 1, 6, 8, and 10 are met
*E*_10_ (1,0,1,0)	*c*_1_−π_2_−π_4_+*s*_3_, Δ*r*_2_−η_2_−*c*_2_+*s*_2_+π_2_, −(−η_3_−Δ*c*_3_+*s*_3_+π_3_), −*c*_44_+*s*_4_+π_4_	× × × ×	Stable when conditions 2, 3, 8, and 4 are met
*E*_11_ (0,1,1,1)	−(*c*_1_+*s*_2_+*s*_3_+*s*_4_), −(Δ*r*_2_−η_2_−*c*_2_), −(Δ*r*_3_−η_3_),	− × × ×	Stable when conditions 6, 8, and 9 are met
*E*_12_ (1,0,1,1)	*c*_1_+*s*_3_+*s*_4_, Δ*r*_2_−η_2_−*c*_2_+*s*_2_, −(−η_3_−Δ*c*_3_+*s*_3_), −(*c*_44_+*s*_4_+π_4_)	+ × × −	Instable
*E*_13_ (1,1,0,1)	*c*_1_+*s*_4_+*s*_2_, −(−η_2_−*c*_2_+*s*_2_), Δ*r*_3_−η_3_+*s*_3_, −(*c*_42_−*c*_43_+*s*_4_+π_4_)	+ × × ×	Instable
*E*_14_ (1,1,0,0)	*c*_1_−π_3_−π_4_+*s*_2_, −(−η_2_−*c*_2_+*s*_2_+π_2_), Δ*r*_3_−η_3_+*s*_3_+π_3_, *c*_42_−*c*_43_+*s*_4_+π_4_	× × × ×	Stable when conditions 11-14 are met
*E*_15_ (1,1,1,0)	*c*_1_−π_4_+*s*_2_+*s*_3_, −(Δ*r*_2_−η_2_−*c*_2_+*s*_2_+π_2_), −(Δ*r*_3_−η_3_+*s*_3_+π_3_), *c*_41_−*c*_43_+*s*_4_+π_4_	× × × ×	Stable when conditions 11-14 and 15-16 are met
*E*_16_ (1,1,1,1)	*c*_1_+*s*_2_+*s*_3_+*s*_4_, −(Δ*r*_2_−η_2_−*c*_2_+*s*_2_), −(Δ*r*_3_−η_3_+*s*_3_), −(*c*_41_−*c*_43_+*s*_4_+π_4_)	+ × × −	Instability

× represents positive and negative uncertainty, the same below. Condition 1: π_2_+π_3_+π_4_<*c*_1_; Condition 2: *c*_1_ < π_2_+π_4_−*s*_3_; Condition 3: Δ*r*_2_−*c*_2_+*s*_2_ < η_2_−π_2_; Condition 4: *s*_3_−Δ*c*_3_ < η_3_−π_3_; Condition 5: *s*_4_−*c*_44_ < −π_4_; Condition 6: Δ*r*_2_−*c*_2_>η_2_; Condition 7: *s*_3_−Δ*c*_3_>η_3_−π_3_; Condition 8: Δ*r*_3_>η_3_; Condition 9: *c*_41_>*c*_43_; Condition 10: *c*_41_<*c*_43_; Condition 11: *c*_1_ < π_4_−*s*_2_−*s*_3_; Condition 12: η_2_−π_2_<*s*_2_−*c*_2_; Condition 13: Δ*r*_3_+*s*_3_ < η_3_−π_3_; Condition 14: *s*_4_−*c*_43_ < −π_4_−*c*_42_; Condition 15: Δ*r*_3_+*s*_3_>η_3_−π_3_; Condition 16: *s*_4_−*c*_43_ < −π_4_−*c*_41_.

#### 5.2.1 Scenario 1

The stable point (0, 0, 0, 0) means that the government supervision department is relaxed, banks and financial institutions implement traditional credit, non-bank financial institutions do not enter, and high carbon enterprises exceed their emissions standards. The stable condition is π_2_+π_3_+π_4_<*c*_1_. At this time, the net regulatory benefit of the regulatory authorities is < 0, and the ultimate strategic choice of the regulatory authorities will tend to be a loose supervision strategy. Banks, non-banking financial institutions, and high-carbon enterprises will all choose methods beneficial to their interests but detrimental to social development by observing the government's plan. This situation is a situation of government inaction and market failure, which is the least ideal situation in reality. Therefore, it is required that the regulatory authorities establish a strict regulatory system, provide specific incentives for banks and non-banking financial institutions, and actively implement national green development policies for high-carbon enterprises. In addition, severe punishment should be imposed when violations of national industrial policy development directions are discovered.

#### 5.2.2 Scenario 2

The stable point (1, 0, 0, 0) means that government regulatory authorities strictly regulate banks and financial institutions to implement traditional credit, non-bank financial institutions actively refuse to enter the green finance market, and high carbon enterprises exceed their emissions standards. The stable conditions are π_2_+π_3_+π_4_>*c*_1_, *s*_3_+π_3_−Δ*c*_3_ < η_3_, *s*_2_−*c*_2_+ < η_2_−π_2_, *s*_4_−*c*_44_ < −π_4_. Compared with scenario 1, the net regulatory benefit of the government regulatory authorities is >0, and the government's strategic choice is gradually inclined toward strict regulatory strategies. However, at this time, the incentive measures are not perfect or limited by the high cost of the other three parties' game subjects themselves, and banking financial institutions will not actively implement green financial policies, non-banking financial institutions will not actively enter the green financial market, and the net benefit of high-carbon enterprises' compliance emissions is less than that of excessive emissions. Therefore, they will insist on choosing the excessive emissions strategy. This situation is a failure of government macro-control; that is, the government's regulatory measures have a small regulatory effect on the market, which is also unsatisfactory. At this time, the regulatory authorities must develop a regulatory system that is more in line with the actual situation of market economic development and promote green economic growth in government macro-control.

#### 5.2.3 Scenario 3

The stable point (1, 0, 0, 0) means (that government regulatory authorities strictly regulate banks and financial institutions to implement traditional credit, non-bank financial institutions actively refuse to enter the green finance market, and high carbon enterprises exceed their emissions standards). The stable condition are *c*_1_>π_4_−*s*_2_−*s*_3_, Δ*r*_2_−*c*_2_>η_2_, Δ*r*_3_>η_3_, *c*_41_<*c*_43_. Compared with scenario 1, the net regulatory benefit of the government regulatory authorities is >0, and the government's strategic choice is gradually inclined toward strict regulatory strategies. However, at this time, the incentive measures are not perfect or limited by the high cost of the other three parties' game subjects themselves, and banking financial institutions will not actively implement green financial policies, non-banking financial institutions will not actively enter the green financial market, and the net benefit of high-carbon enterprises' compliance emissions is less than that of excessive emissions. Therefore, they will insist on choosing the excessive emissions strategy. This situation is a failure of government macro-control; that is, the government's regulatory measures have a small regulatory effect on the market, which is also unsatisfactory. At this time, the regulatory authorities must develop a regulatory system that is more in line with the actual situation of market economic development and promote green economic growth in government macro-control.

#### 5.2.4 Scenario 4

The stable point (1, 0, 1, 0) means (government regulatory authorities strictly supervise, bank financial institutions execute traditional credit, non-bank financial institutions actively enter the green finance market, and high carbon enterprises exceed their emissions standards). The stable conditions are π_2_+π_4_−*s*_3_>*c*_1_, Δ*r*_2_−*c*_2_+*s*_2_ < η_2_−π_2_, *s*_3_−Δ*c*_3_>π_3_−η_3_, and *s*_4_−*c*_44_ < −π_4_. Compared to scenario 2, at this time, as non-banking financial institutions enter the green financial market, the net benefit is greater than that of not entering, and the strategy choice tends to enter the green financial market actively. However, the strategy choices of banks and high-carbon enterprises are still relatively passive. Currently, the government's regulatory system still needs to be further improved. To promote the development of the green credit market, it is necessary to focus on the execution of banking and financial institutions, supplemented by non-bank financial institutions. Similarly, high-carbon enterprises are limited by high transformation costs and still adhere to the strategy of excessive emissions. Therefore, the current situation still needs improvement and requires further macroeconomic regulation by the government to promote green and healthy economic development.

#### 5.2.5 Scenario 5

The stable point (0, 1, 1, 1) means (government regulatory authorities loosely regulate, bank financial institutions implement green credit, non-bank financial institutions actively enter the green finance market, and high-carbon enterprises meet emission standards). The stable condition are Δ*r*_2_−*c*_2_>η_2_, Δ*r*_3_>η_3_, and *c*_41_>*c*_43_. This situation is in a relatively ideal state; at this time, the regulatory authorities do not need to take strict regulatory measures, and the market economy will form a virtuous cycle. The banking financial institutions actively respond to the national industrial policy, implement green credit policies, and pass on the green concept to the entire financial system. Non-banking financial institutions are also driven by the bank's signal effect and actively enter the green market. At this time, not only will the green financial market develop significantly, but it will also play a more critical role in promoting high-quality economic development. With the rapid growth of the green financial market, high-carbon enterprises are also more likely to receive support from green credit. At this time, the increasing maturity of transformation technology and the reduction of capital costs will ultimately encourage high-carbon enterprises to choose the emission standards strategy.

#### 5.2.6 Scenario 6

The stable point (1, 1, 0, 0) means (government regulatory authorities strictly supervise, bank financial institutions implement green credit, non-bank financial institutions actively refuse to enter the green finance market, and high-carbon enterprises exceed their emissions standards). The stable conditions are *c*_1_ < π_3_+π_4_−*s*_2_, η_2_−π_2_<*s*_2_−*c*_2_, Δ*r*_3_+*s*_3_ < η_3_−π_3_, and *s*_4_−*c*_43_ < −π_4_−*c*_42_. This state is the condition of government and financial malfunction markets, where the government actively supervises and banking financial institutions actively implement green credit policies. Still, the effect is poor, and the signal effect fails within the financial system. The government regulatory authorities also fail to exert their due deterrent power. This requires the government regulatory authorities to increase the punishment of non-banking financial institutions that do not enter the green financial market and high-carbon enterprises that exceed emission standards to avoid the emergence of stable states that are not conducive to high-quality socio-economic development.

#### 5.2.7 Scenario 7

The stable point (1, 1, 1, 0) means (government regulatory authorities strictly supervise, bank financial institutions implement green credit, non-bank financial institutions actively enter the green finance market, and high carbon enterprises exceed their emissions standards). The stable conditions are *c*_1_ < π_4_−*s*_2_−*s*_3_, Δ*r*_2_−*c*_2_+*s*_2_>η_2_−π_2_, Δ*r*_3_+*s*_3_>η_3_−π_3_, and *s*_4_−*c*_43_ < −π_4_−*c*_41_. Compared to scenario 6, non-banking financial institutions choose to actively enter the green financial market because the net income from entering the green financial market is more significant than zero. However, at this time, high-carbon enterprises still choose to emit excessively, indicating that relying solely on the development of the green financial market has not driven the green transformation of high-carbon enterprises. The high transformation costs of high-carbon enterprises remain their primary consideration. This requires government regulatory authorities to intensify rewards and punishments further, and banks and non-banking financial institutions should also further invest in the green financial market to promote their green transformation to reduce enterprises' transformation costs.

## 6 Numerical simulation analysis

To more intuitively describe the behavior strategy selection of multiple participating subjects in the green finance process promoting corporate carbon emission reduction, we will use MATLAB tools for numerical simulation analysis, focusing on the evolution of strategic choices among government regulatory agencies, bank financial institutions, non-bank financial institutions, and high-carbon enterprises.

### 6.1 Numerical simulation analysis under ideal strategy combination

To more intuitively reflect the evolutionary path of the four-game players of government regulatory authorities, bank financial institutions, non-bank financial institutions, and high-carbon enterprises, this study chooses to introduce the theory of willingness behavior into simulation analysis based on the stable point (0, 1, 1, 1). It examines the impact of the initial willingness of the game players and changes in relevant parameters on the stable state of the four players' strategy selection. Based on this, this article refers to the practices of Sun and Qu (10) and Cui et al. (36) and assign the relevant parameters based on the parameter constraints of scenario five as follows: *c*_1_ = 4, π_2_ = 3, π_3_ = 4, π_4_ = 5, *s*_2_ = 3, *s*_3_ = 4, *s*_4_ = 5, Δ*r*_2_ = 10, η_2_ = 3, *c*_2_ = 1, Δ*c*_3_ = 2, Δ*r*_3_ = 10, η_3_ = 2, *c*_41_ = 10, *c*_42_ = 4, *c*_43_ = 3, *c*_44_ = 2.

#### 6.1.1 The impact of the initial willingness of government regulatory bodies on the equilibrium results

**Figure 6:**
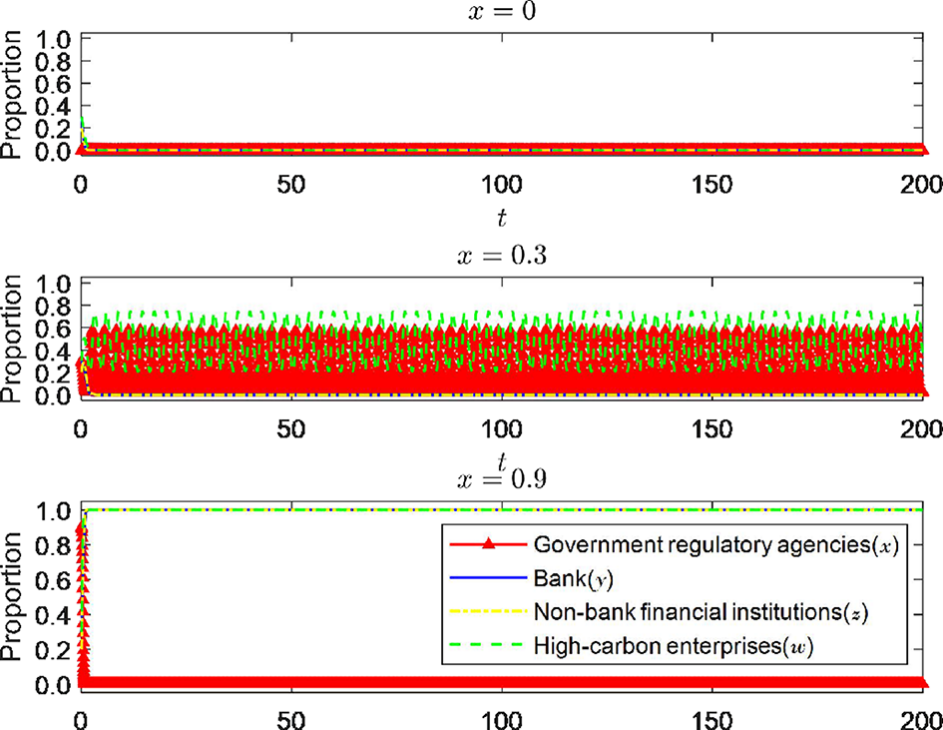
The impact of changes in the initial willingness *x* of government regulatory agencies.

As the leading force in promoting carbon reduction through green finance, government regulatory agencies will significantly impact the strategic choices of the other three parties in the game. This article attempts to embed the theory of willingness behavior into evolutionary game analysis, considering the impact of one party's initial willingness on the strategy choices of other parties and the direction of system evolution. According to Figure 6, when the initial desire of the government regulatory authority is 0, that is, when it is left to its own devices and does not carry out any supervision, the strategic choices of banks and non-bank financial institutions, as well as high-carbon enterprises, tend to evolve in a direction that is detrimental to economic and social development. Banks do not implement green credit policies, non-bank financial institutions do not enter the green financial market, and high-carbon enterprises choose to exceed emission standards. This is the most unfavorable situation. Of course, the government regulatory authority will take measures to avoid severe social losses. When the initial willingness of the government regulatory authority to supervise increases to 0.3, the strategic choices of the other three parties in the game do not change in the same direction. The evolution speed of the strategic decisions of banks and non-bank financial institutions slows down. However, they must still implement green credit policies and enter the green financial market. The possible reason is that, firstly, green finance plays a vital role in pursuing high-quality economic development as a boosting tool. Still, banks and non-bank financial institutions are not the focus of government supervision. The slight increase in supervision strength should mainly affect high-carbon enterprises. At this time, whether high-carbon enterprises meet emission standards will directly affect the pursuit of high-quality economic development. Therefore, after a short period of fluctuation, they will still choose strategies more conducive to their development. At this time, the high-carbon enterprises are affected by the increase in supervision strength, and their strategic choices will change with the change in the supervision strength of the government regulatory authority. This trend can also be seen in the figure. When the initial willingness of the government regulatory authority to supervise further increases to 0.9, the system will evolve to an ideal stable state (0, 1, 1, 1); that is, at this time, the government regulatory authority does not need to spend more energy on supervision, and the other three parties in the game will also evolve to a stable state that is conducive to economic development. Banks and non-bank financial institutions will actively implement green credit policies and enter the green financial market. High-carbon enterprises will also choose to meet emission standards. It can be seen that the linkage effect between the wills of the government regulatory authority and the other three parties in the game, especially high-carbon enterprises, further proves the existence of the influence of the theory of willingness behavior in the evolutionary game process and also expands the application of evolutionary game theory to a certain extent.

#### 6.1.2 The impact of green credit signal effect

**Figure 7:**
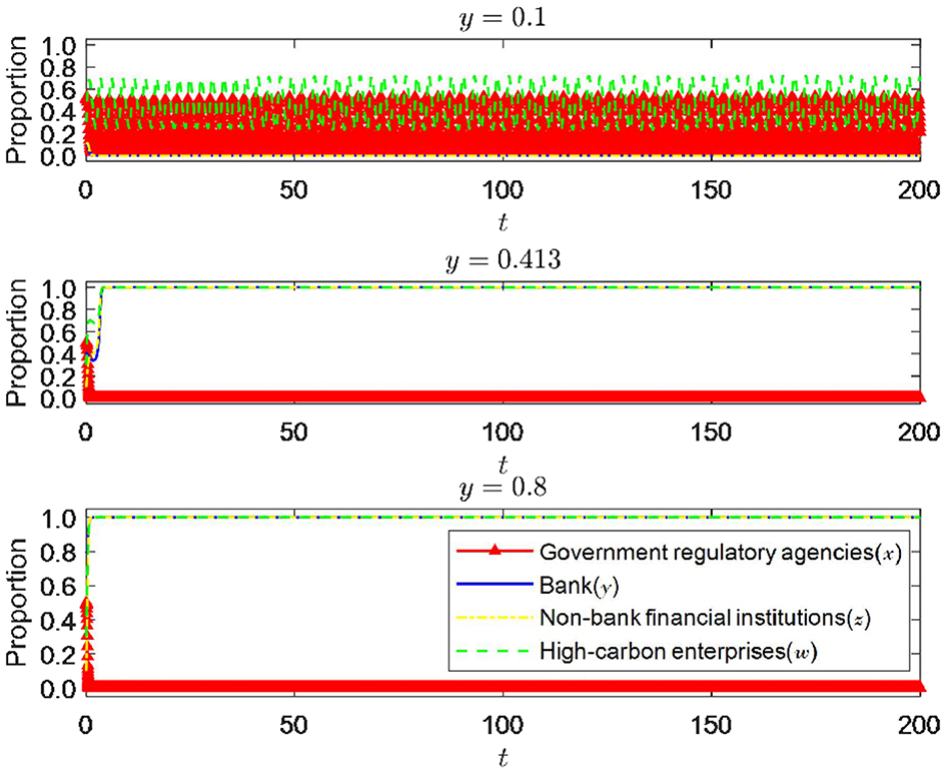
The impact of changes in initial willingness of banks and financial institutions.

As an essential support for the entire financial system, banking financial institutions have always played an important role in developing the green financial market. By actively implementing green credit policies, banks can transmit the concept of green development throughout the financial system. As shown in Figure 7, when the initial willingness of banking financial institutions increases from 0.1 to 0.413 while the initial desire of government regulatory departments, non-bank financial institutions, and high-carbon enterprises remains unchanged (*x* = 0.5, *z* = 0.1, *w* = 0.3), the strategic choices of all parties undergo significant changes. When the initial willingness of bank financial institutions is 0.1, non-bank financial institutions also tend to choose not to enter the green financial market. Currently, the strategic choices of government regulatory departments and high-carbon enterprises fluctuate and do not form a unified trend. The reason may be that government regulatory departments consider that the green development willingness of all parties in the green financial market is low, so their strategic choices are always in a state of strict supervision and loose supervision. For high-carbon enterprises, meeting emission standards involves high transition costs brought about by green transformation. Therefore, the primary considerations for government regulatory departments are their reward and punishment measures and whether banking and financial institutions can provide sufficient green financial support. Thus, regarding unclear strategic choices among all parties, high-carbon enterprises will always fluctuate regarding whether to undergo green transformation. Non-bank financial institutions, when the initial willingness of banking financial institutions is low, also tend to choose not to enter the green financial market. When the initial desire of banking and financial institutions rises to 0.413, all parties' strategic choices tend to be more precise and eventually toward an ideal stable state. Currently, government regulatory departments do not need strict supervision, and banking and financial institutions will actively implement green credit. Affected by green development, non-bank financial institutions will also actively enter the green financial market, and high-carbon enterprises will choose to undergo green transformation and meet emission standards. When the initial willingness of bank financial institutions continued to rise to 0.8, all parties' strategic choices tended to be more precise and eventually tended toward an ideal stable state. However, compared to when the initial willingness is 0.413, the convergence speed is significantly faster, which means that all parties will converge toward an ideal stable state faster. Through the above analysis, it is further verified that there is a signal effect in the green financial market, and this signal effect will gradually increase as the initial willingness increases.

#### 6.1.3 The impact of changes in subsidy intensity of government regulatory departments

**Figure 8:**
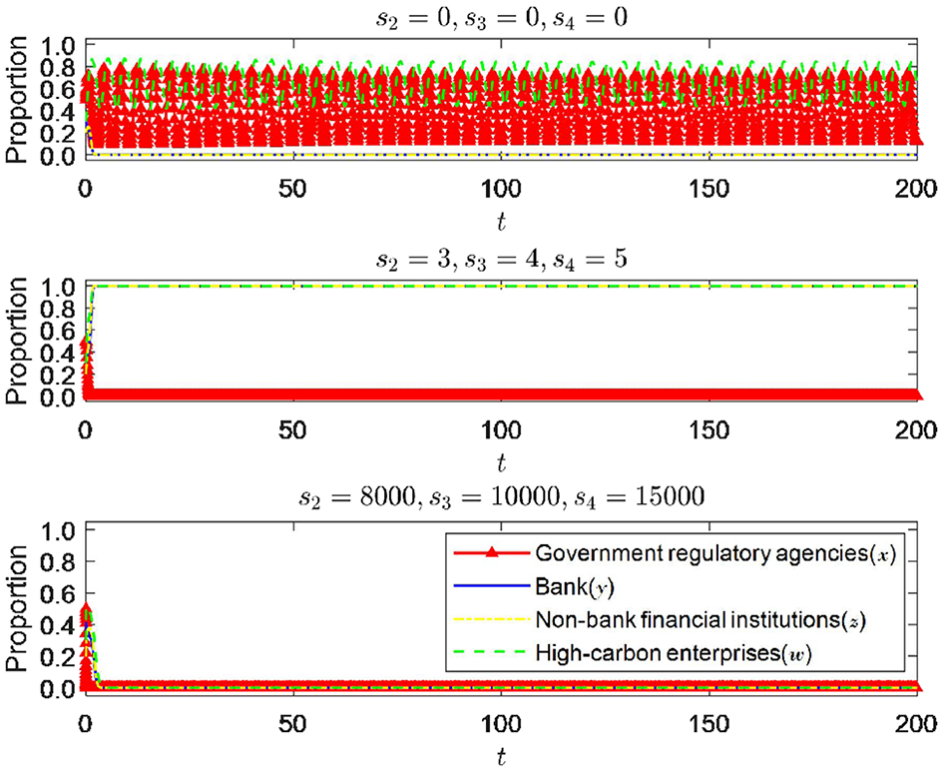
The impact of changes in subsidy intensity.

It can be seen from Figure 8 that when other parameters are fixed, different subsidy levels significantly impact the system's equilibrium state. When the government regulatory authorities do not take subsidy measures, that is, *s*_2_ = *s*_3_ = *s*_4_ = 0, the strategic choices of banks and non-bank financial institutions tend not to implement green credit policies and not enter the green financial market. However, high-carbon enterprises are constrained by the high cost of green transformation and must also consider the more severe consequences of exceeding emission standards. Under dual pressures, it is difficult for high-carbon enterprises to form stable strategies. Since the government regulatory authorities' strategic choices fluctuate and do not form stable strategies at this time, high-carbon enterprises also do not create a unified strategy choice with their fluctuations. When the subsidy intensity of regulatory authorities is further increased, that is, increased to 3, 4, and 5, respectively, the system tends to reach an ideal stable state, indicating that the subsidy measures of regulatory authorities will have a significant impact on the strategic choices of all parties involved. This impact will gradually increase as the subsidy level increases. However, government subsidies are not sustainable, and both the high-quality development of financial markets and the green transformation of high carbon enterprises must rely on the strength of each entity itself. From the graph, it can be seen that when the subsidy intensity increases without limit, the final effect achieved is that the system tends toward the least ideal stable point of (0, 0, 0, 0), which means that the promotion effect of subsidy intensity on other entities has a marginal decrease. Regulatory authorities need to control the length and width of subsidy policies in order to achieve the optimal subsidy effect.

#### 6.1.4 The impact of changes in the intensity of punishment by government regulatory authorities

**Figure 9:**
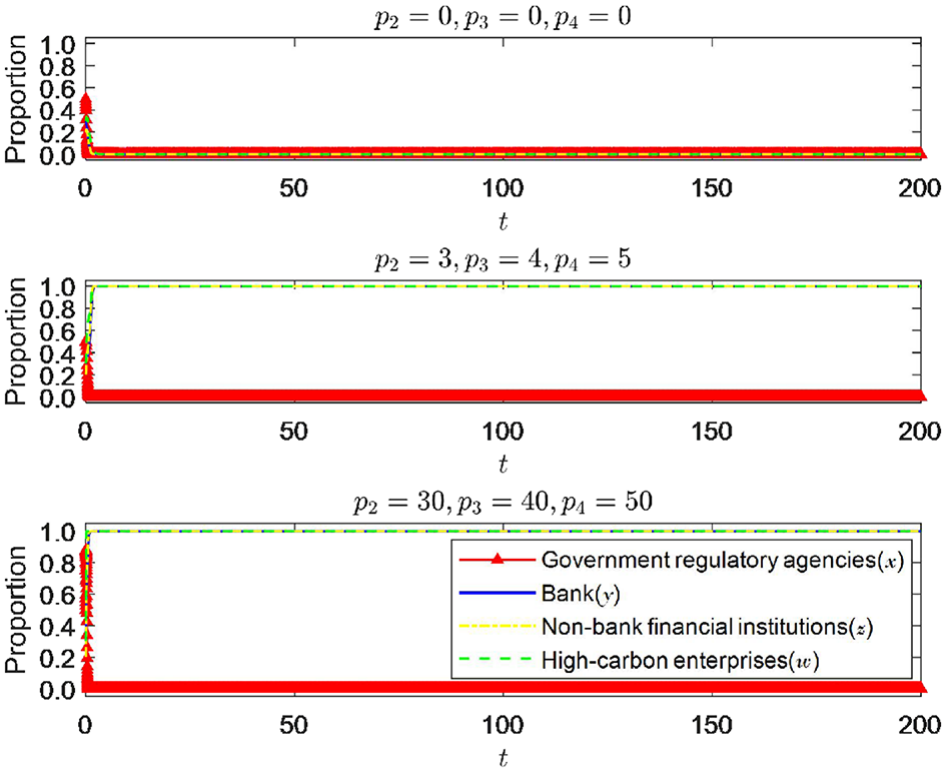
The impact of changes in punishment intensity.

As shown in Figure 9, *p*_2_, *p*_3_, *p*_4_ instead of π_2_, π_3_, π_4_, when other parameters are fixed, and the punishment intensity is changed, the change in punishment intensity significantly impacts the equilibrium state of the system. When the punishment intensity is 0, it can be seen that the system eventually approaches (0, 0, 0, 0), which is the minor ideal state and highly detrimental to economic and social development. It is a state that needs to be avoided. It can be seen that the deterrent effect of the punishment intensity of government regulatory authorities on all parties is essential and will significantly impact the system's stability. When the punishment intensity increased to 3, 4, and 5, respectively, the system approached the ideal state of (0, 1, 1, 1). Therefore, it can be seen that the punishment intensity of regulatory authorities also needs to reach a certain threshold to be more effective. However, according to Figure 9, as the punishment intensity of government regulatory departments further increases, the evolution trend of the strategy choices of the four parties remains almost unchanged, indicating that the further increase in punishment intensity will not have any further impact on the strategy choices of all parties. It can be inferred that when the punishment intensity exceeds the threshold, there is a diminishing marginal effect of further increasing the punishment intensity on the regulatory effect. Therefore, regulatory authorities are required to measure the punishment intensity well to ensure the optimal punishment regulatory effect.

#### 6.1.5 The impact of changes in the price difference between traditional credit and green credit income of banking financial institutions

**Figure 10:**
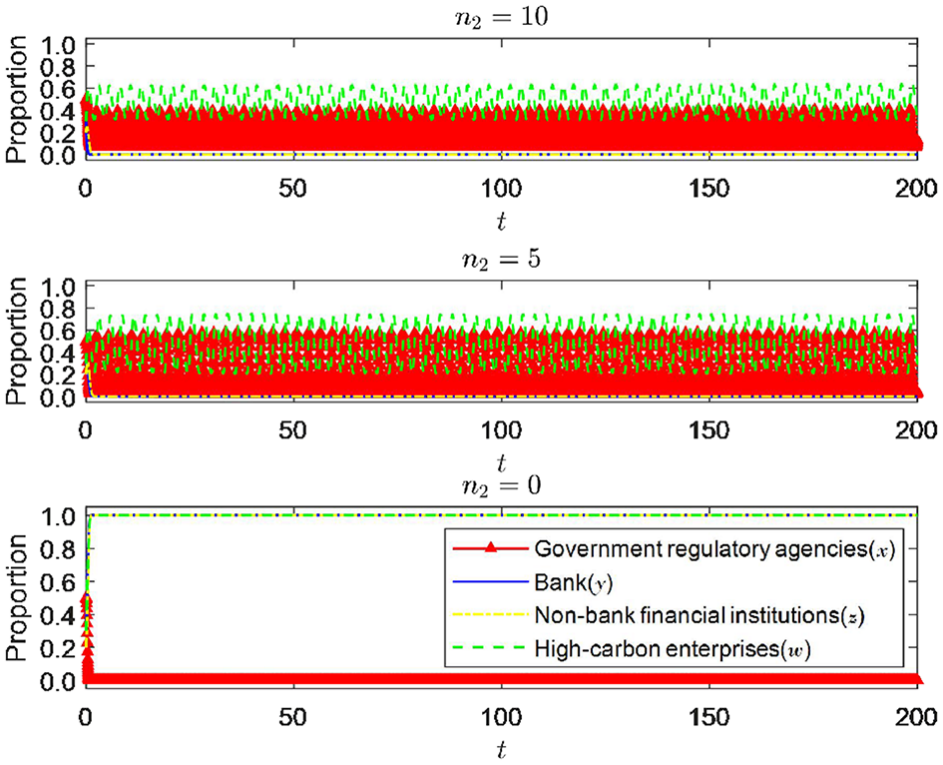
Impact of changes in yield spread between traditional credit and green credit.

As shown in Figure 10, while substituting *n*_2_ for η_2_ and other parameters remaining unchanged, the change in the return spread significantly impacts the system's equilibrium state. When *n*_2_ is 10, traditional credit's return is significantly higher than green credit's, and banks will choose traditional credit. Non-bank financial institutions are also affected by the bank's strategic choice and eventually tend not to enter the green financial market. At this time, government regulatory authorities, due to their inability to drive banks and non-bank financial institutions into the green financial market and thus promote the green transformation of high-carbon enterprises, will fluctuate in choosing strict or loose regulation and ultimately not form a stable strategic choice. High-carbon enterprises are also affected by fluctuations in the government regulatory authorities' strategic decisions, and their strategies are unstable. When *n*_2_ is 5, the return spread becomes more minor. However, because the return on traditional credit is still higher than that of green credit, banks still choose traditional credit driven by profit-seeking attributes. The strategic choices of government regulatory authorities and high-carbon enterprises are still fluctuating and not stable during the process. Finally, when *n*_2_ is 0, that is, there is no return spread between traditional credit and green credit, it is clear that banks and non-bank financial institutions will consider their social attributes more on the basis of ensuring their own returns, and their strategic choices will eventually converge toward implementing green credit policies and actively entering the green financial market. In this case, the green transformation of high-carbon enterprises can receive sufficient green financial support, significantly reducing the transformation costs of enterprises, and high-carbon enterprises will eventually stabilize under the emission compliance strategy. Currently, the regulatory authorities do not need strict supervision, and the system will tend toward an ideal stable state.

#### 6.1.6 Impact of changes in risk-return of non-bank financial institutions

**Figure 11:**
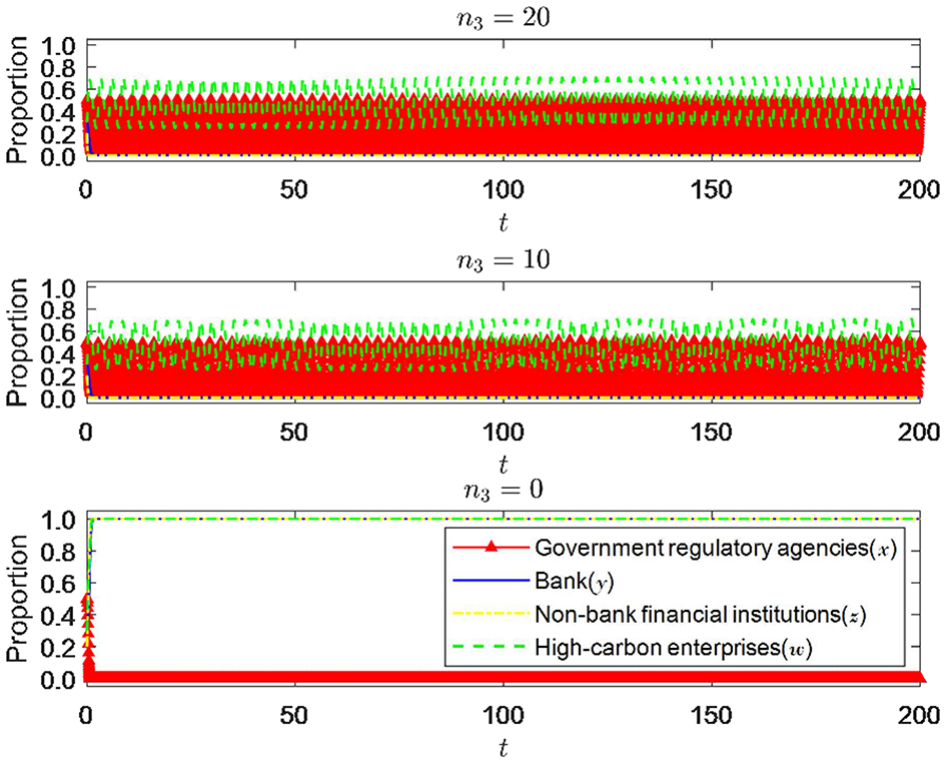
Impact of risk return changes on non-bank financial institutions.

As shown in Figure 11, substituting *n*_3_ for η_3_ and other parameters remaining unchanged, the change in risk-return of non-bank financial institutions significantly impacts the system's equilibrium state. The changes in the strategic choices of government regulatory authorities and high-carbon enterprises are similar to those caused by the difference above in the return on traditional credit and green credit implemented by banks and are not discussed in detail here. It should be emphasized that the signal-effect transmission between banks and non-bank financial institutions is mutual. As can be seen from the figure, with the gradual decrease in risk-return, the strategic choices of non-bank financial institutions have shifted from not entering the green financial market to eventually entering it. In this process, the strategic decisions of bank financial institutions have also shifted from choosing traditional credit strategies to eventually choosing green ones. It can be seen that the green concept of non-bank financial institutions will gradually be transmitted to bank financial institutions and have a particular impact on their strategic choices.

#### 6.1.7 Impact of cost changes on high-carbon enterprises

**Figure 12:**
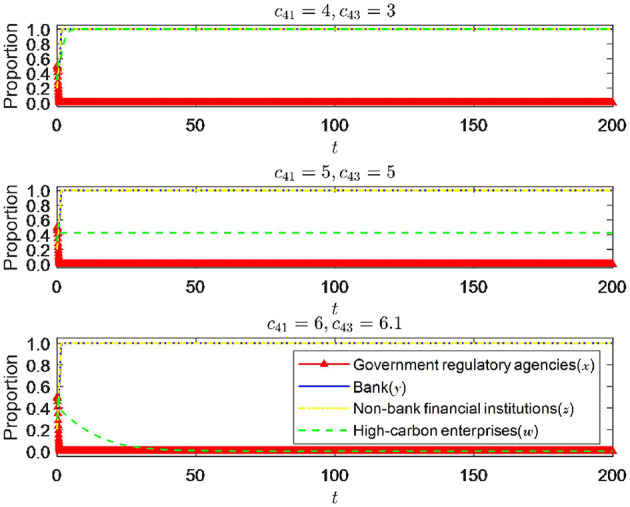
Impact of cost changes on high carbon enterprises.

It can be seen from Figure 12 that when other parameters are fixed, the main factor considered for whether high-carbon enterprises meet emission standards is still their costs. When *c*_41_ and *c*_43_ are 4 and 3, respectively, it can be understood that the cost of exceeding emission standards is higher than that of meeting emission standards, and high-carbon enterprises will choose to meet emission standards. Banks and non-bank financial institutions are also more willing to provide green financial support for high-carbon enterprises. In this case, the regulatory authorities will slightly relax their supervision efforts, and the system will approach an ideal state. When *c*_41_ = *c*_43_ = 5, the cost of meeting emissions standards equals the cost of exceeding emissions. The strategy choices of high-carbon enterprises are constant and not biased.

On the one hand, exceeding emissions may be punished by regulatory authorities, and on the other hand, meeting emissions standards does not bring more benefits to high-carbon enterprises. Therefore, the strategy choices of high-carbon enterprises will remain constant compared to their initial willingness. When *c*_41_ and *c*_43_ are 6 and 6.1, respectively, the cost of exceeding emission standards is slightly lower than that of meeting emission standards; it can be seen that the strategic choice of high-carbon enterprises will eventually approach that of exceeding emission standards. At this time, there is a drift toward green behavior in high-carbon enterprises, and the development of green financial markets has not effectively promoted the green transformation of high-carbon enterprises.

#### 6.1.8 The impact of the bank's green credit policy on high-carbon enterprises

**Figure 13:**
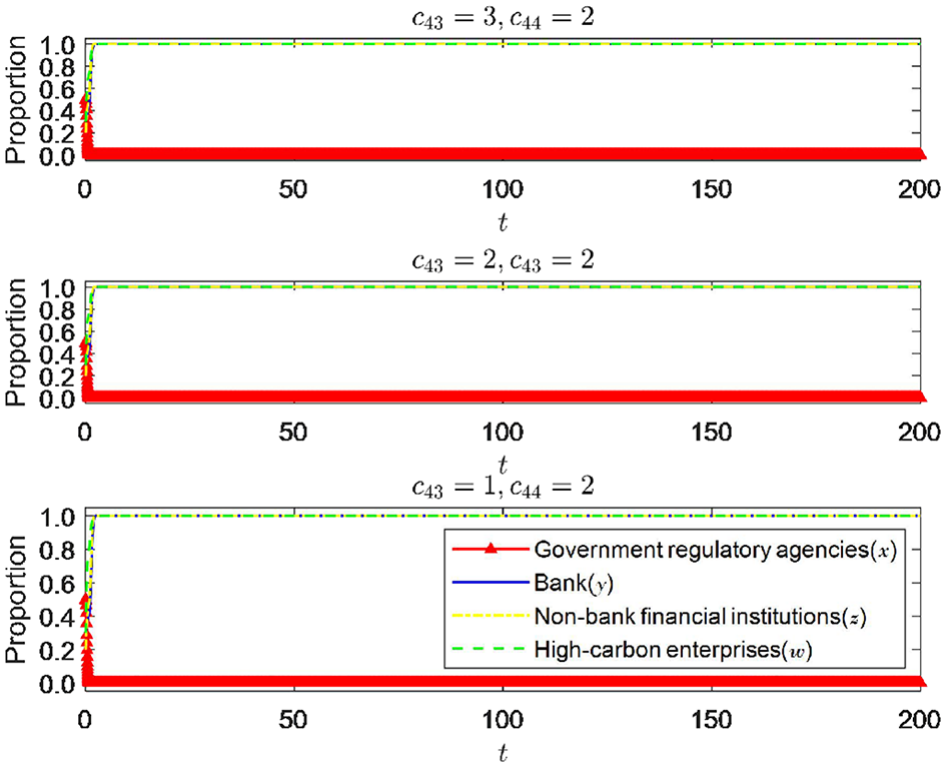
Sensitivity of high carbon enterprises to green credit policies of banks.

As shown in Figure 13, when the values of *c*_43_ and *c*_44_ are adjusted in any way, the strategic choices of all parties tend to be ideal and stable. It can be seen that the degree of willingness of high-carbon enterprises to choose their strategies is more critical than their strategic choices. When high-carbon enterprises actively choose green transformation, the regulatory authorities do not need to intervene too much. Whether banks and non-bank financial institutions provide green funds will not change the strategic choices of high-carbon enterprises. Of course, banks and non-bank financial institutions are more willing to provide green transformation funds for high-carbon enterprises with strong environmental awareness. While pursuing their interests, they also actively fulfill their social responsibilities. It can be seen that when high-carbon enterprises have solid ecological awareness, they are not sensitive to the green credit policies of banks. On the contrary, if high-carbon enterprises have weak transformation willingness, they may be more sensitive to green funding support from banks and non-bank financial institutions.

#### 6.1.9 Analysis of the impact of government regulatory mechanisms on the strategic choices of all parties

**Figure 14:**
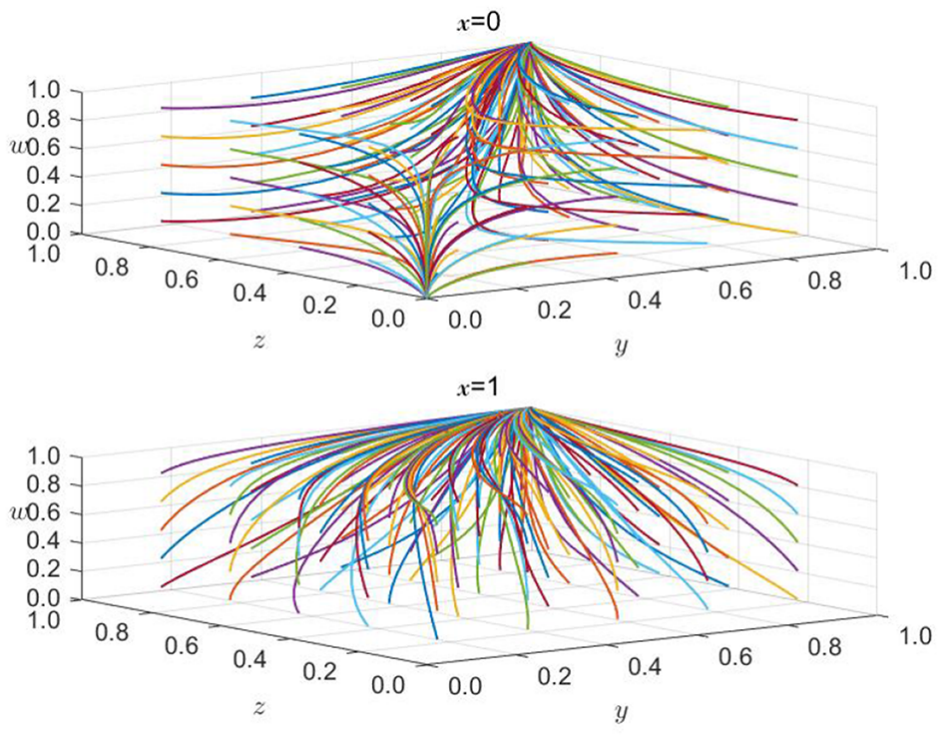
Analysis of the impact of government regulatory mechanisms on strategic choices of various parties.

Figure 14 shows that when *x* = 0, there is no unique stable strategy for the strategy selection of all parties. Currently, there are two stable strategies, (0, 1, 1, 1) and (0, 0, 0, 0), which are the most ideal and least ideal stable strategies. The development of the green financial market and its assistance in reducing carbon emissions from high-carbon enterprises require macroeconomic regulation and control from the government to make the system eventually approach an ideal stable state. When *x* = 1, when the government regulatory department chooses a strict regulatory strategy, there is only one stable strategy choice (1, 1, 1, 1) in the system, which further verifies the effectiveness of the government regulatory department's macroeconomic regulation and control mechanism. Similarly, banks and non-bank financial institutions will actively promote the development of the green financial market, further promoting the green transformation of high-carbon enterprises, thus forming a virtuous cycle of economic growth and ultimately achieving high-quality economic development.

#### 6.1.10 Analysis of the impact of green credit policies on the strategic choices of various stakeholders

**Figure 15:**
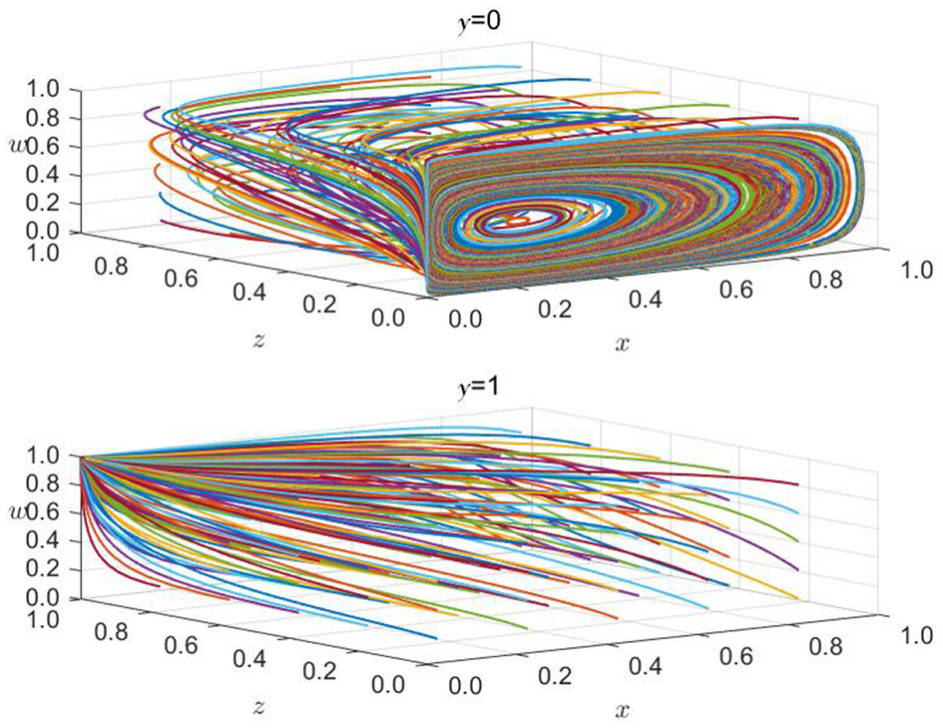
Analysis of the impact of green credit policies on the strategic choices of various parties.

As shown in Figure 15, when *y* = 0, that is, when the bank financial institution does not implement the green credit policy and chooses the traditional credit strategy, the system tends to (0, 0, 0, 0, 0). However, it can be seen from the figure that this trend is not stable. Therefore, it is necessary to change the bank's strategy selection to change this trend. When *y* = 1, that is, when the bank financial institution chooses the green credit policy, there is a unique stable strategy (0, 1, 1, 1) in the system. It can be seen that banks, as the main driving force for the development of the green financial market, will directly affect other entities. Among them, non-bank financial institutions are affected by signal effects, and due to their access to more market information, they need to pay additional costs to enter the green financial market without implementing the green credit policy. Therefore, non-bank financial institutions will not choose to enter the green financial market. When a bank or financial institution implements the green credit policy, it is affected by signal effects and the reduction of information costs. Now, non-bank financial institutions will also actively enter the green financial market.

Similarly, high transition costs are the main obstacle to the green transformation of high-carbon enterprises. Because banks and non-bank financial institutions do not implement the green credit policy, high-carbon enterprises cannot obtain sufficient low-cost financial support, so they prefer to risk excessive emissions rather than green transformation. When high-carbon enterprises can obtain sufficient green financial support, they will actively carry out green transformation and ultimately achieve emission compliance. In the above cases, regulatory authorities can gradually relax their supervision efforts, and the green financial market will develop significantly. Energy conservation and emission reduction efforts by high-carbon enterprises will also make some progress.

## 7 Conclusions

The ultimate goal of high-quality development is coordinating economic growth and environmental sustainability. The development of green financial markets and the green transformation of high-carbon enterprises are significant for achieving high-quality economic development. Based on this, this article constructs a four-party evolutionary game model of government regulatory departments, banks, non-bank financial institutions, and high-carbon enterprises to explore the changes in the strategic choices of all parties in the process of green finance assisting enterprises in carbon emission reduction, as well as the constraints that need to be met to achieve the desired state. Through analysis, the following conclusions and suggestions are drawn:

(1) The reward and punishment mechanisms of government regulatory authorities significantly impact the strategic choices of the other three parties in the game. Increasing subsidies and penalties will promote the system to evolve to an ideal stable state. Still, there is a threshold for the intensity of rewards and punishments and a phenomenon of diminishing marginal benefits in their effects. This requires the regulatory authorities to consider this phenomenon fully when formulating the reward and punishment system. Excessive rewards and punishments will reduce effectiveness, while insufficient rewards and punishments will not achieve the desired deterrent effect. In addition, according to the theory of voluntary behavior and simulation analysis, there is a linkage effect between the strategic choices of government regulatory authorities and high-carbon enterprises; that is, the strategic decisions of regulatory authorities will have an important impact on the voluntary choices of high-carbon enterprises. The greater the intensity of supervision by regulatory authorities, the greater the risk faced by high-carbon enterprises in exceeding emission standards, which is of great significance for the green transformation of high-carbon enterprises. Therefore, it is required that the regulatory authorities formulate a more scientific and reasonable reward and punishment system to maximize their essential role in achieving high-quality economic development. In the early stages of development, stricter regulatory measures should be implemented to guide the rapid growth of the green financial market while actively cultivating the green concept of high-carbon enterprises to promote their green transformation. Later, the intensity of supervision should gradually weaken. It is now more important to actively guide banks and non-bank financial institutions to implement green financial policies to promote the green transformation of high-carbon enterprises. This is also an ideal stable state in which the role of government regulatory authorities is even more critical. Specifically, it includes: firstly, improving the green finance standard system, clarifying the boundaries of transformational finance, such as developing differentiated industry transformation standards, establishing corporate carbon accounts and rating mechanisms, etc. Secondly, it is necessary to strengthen policy incentives and constraints, establish market-oriented transformation mechanisms, such as setting up special incentive funds and risk compensation mechanisms, implementing differentiated regulatory assessments, etc. Finally, it is necessary to strengthen environmental information disclosure and capacity building, reduce the cost of “green awareness,” such as building a unified environmental information sharing platform, conducting pilot projects for transformational finance, and promoting experience.

(2) The signal effect exists in the green finance system, and the strategic choices of banks and non-bank financial institutions have mutual influences. When banks and financial institutions with information advantages are strongly willing to implement green credit, they will guide non-bank financial institutions to actively enter the green finance market, which will promote the development of the green finance market and the carbon emission reduction behavior of high-carbon enterprises. Therefore, it is required that bank financial institutions continue to play their advantages in market information and other aspects, play a demonstration and leading role in the green finance market, maintain the pertinence and stability of the implementation of green credit policies, and actively encourage non-bank financial institutions to enter the green finance market. Promoting the innovation of green finance products can make up for the shortage of green credit fund supply, thus promoting the development of the green finance market and providing sufficient financial support for the green transformation of high-carbon enterprises to achieve high-quality economic development.

(3) Simulation analysis shows that the high transformation costs faced by high-carbon enterprises are still the main reason for their choice of excessive emissions. Compared with external factors such as the reward and punishment measures of regulatory authorities and the green financial support of financial institutions, the willingness of high-carbon enterprises to transform themselves is more important. At the same time, for the green transformation of high-carbon enterprises, government regulatory authorities' reward and punishment measures still play a leading role. In contrast, green financial policies should be more complementary to government regulatory policies, especially when the green concept of high-carbon enterprises is already strong, the government regulatory authorities will gradually relax supervision, and the role of green finance will become increasingly important. Therefore, to promote the green transformation development of high-carbon enterprises, the main goal should be to reduce their transformation costs, mainly through providing higher transformation subsidies by government regulatory authorities, providing sufficient low-cost green funds by banks and non-bank financial institutions, improving awareness of green transformation among enterprises, and promoting technological innovation in transformation. In addition, it is required that the regulatory authorities reasonably combine their own reward and punishment measures with the green financial strategies of financial institutions to maximize the efficiency of social resource utilization.

Driven by global climate governance and the “dual carbon” goals, green finance has become a core tool for promoting low-carbon transformation in high carbon industries. Research is conducted by constructing a four-party evolutionary game model involving government, banks, non-bank financial institutions, and high carbon enterprises. Firstly, this model can simulate the strategy adjustment paths of various agents under different initial conditions, revealing the dynamic balance of multi-agent strategy selection, which is closer to the gradual transformation process in reality. Secondly, the model can quantify the threshold effects of parameters such as central and local government regulatory strength, financial institution risk preferences, and enterprise emission reduction costs. Such quantitative results provide accurate basis for policy design and avoid the negative effects of “one size fits all” regulation. Finally, this study contributes to optimizing the allocation of green financial resources and policy coordination, further verifying the effectiveness of the “incentive punishment supervision” triple mechanism.

The four party evolutionary game model provides an important framework for understanding the role of green finance in carbon reduction, but it requires researchers to be more realistic in modeling complexity: by introducing heterogeneity, dynamic feedback, and external shocks, the “ideal model” can be transformed into a “decision-making tool” that can guide practice. Future research will further combine behavioral economics experiments and field research data to construct a “theory data policy” closed loop, promoting the transformation of green finance from “scale expansion” to “quality improvement.” In addition, when identifying the game participants in this paper, non-bank financial institutions were not further categorized into specific institutions such as insurance, securities, and funds, which simplified the complexity of the study to some extent. At the same time, due to the unique characteristics of different institutions, a completely homogenized classification may introduce certain errors into the research. In future research, it is hoped that the current deficiencies can be addressed to improve relevant academic research.

## Data Availability

The original contributions presented in the study are included in the article/Supplementary material, further inquiries can be directed to the corresponding author.
