# Supplementary material for: Game analysis of green finance assisting enterprises in carbon reduction under the participation of four parties
Source: Front Public Health. 2025 Sep 1;13:1641134. doi: 10.3389/fpubh.2025.1641134 (PMC12434104; doi:10.3389/fpubh.2025.1641134)
Supplement: Supplementary file 1 [file Data_Sheet_1.pdf]

```
function
dydt=sifang(t,y,c1,t4,t3,t2,s4,s3,s2,ar2,n2,c2,ac3,ar3,n3,c41,c42,c43,c44)
dydt=zeros(4,1);
dydt(1)=y(1)*(y(1) - 1)*(c1-t2-t3-t4+(s4+t2+t3+t4)*y(4)+(s2+t2)*y(2)+(s3+t3)*y(3)-t2*y(2)*y(4)-
t3*y(3)*y(4));
dydt(2)=-y(2)*(y(2) - 1)*(y(3)*ar2-n2-c2+(s2+t2)*y(1)-t2*y(1)*y(4));
dydt(3)=-y(3)*(y(3) - 1)*((ac3+ar3)*y(2)-n3-ac3+(s3+t3)*y(1)-t3*y(1)*y(4));
dydt(4)=-y(4)*(y(4) - 1)*((c42-c43+c44)*y(2)-c44+(s4+t4)*y(1)+(c41-c42)*y(2)*y(3));
end
```

Figure(6)

```
clc;clear;
c1=4,t4=5,t3=4,t2=3,s4=5,s3=4,s2=3,ar2=10,n2=3,c2=1,ac3=2,ar3=10,n3=2,c41=10,c42=4,c43=3,c4
4=2;
%subplot(3,1,1)
set(0,'defaultfigurecolor','w')
[t,y]=ode45(@(t,y)
sifang(t,y,c1,t4,t3,t2,s4,s3,s2,ar2,n2,c2,ac3,ar3,n3,c41,c42,c43,c44),[0,200],[0,0.3,0.2,0.3]);
points=1:1:length(t);
plot(t,y(:,1),'r^-','linewidth',1,'markersize',3,'markerfacecolor','r','markerindices',points); hold on
plot(t,y(:,2),'b-','linewidth',1);
hold on
plot(t,y(:,3),'y-.','linewidth',1);
hold on
plot(t,y(:,4),'g--','linewidth',1);
hold on
set(gca,'XTick',[0:50:200],'YTick',[0.0:0.2:1.0])
set(gca,'YTickLabel',num2str(get(gca,'YTick')','%.1f')); axis([0
200 -0.05  1.05])
xlabel('$t$','interpreter','latex');
ylabel('Proportion');
zhuti=title('$x}=0$'); set(zhuti,'interpreter','latex')
legend(Government regulatory agencies({\it\fontname{BodoniMT}x})','Bank
({\it\fontname{BodoniMT}y})','Non-bank financial institutions({\it\fontname{Bodoni MT}z})','High
-carbon enterprises({\it\fontname{Bodoni MT}w})');

c1=4,t4=5,t3=4,t2=3,s4=5,s3=4,s2=3,ar2=10,n2=3,c2=1,ac3=2,ar3=10,n3=2,c41=10,c42=4,c43=3,c4
4=2;
%subplot(3,1,1)
set(0,'defaultfigurecolor','w')
[t,y]=ode45(@(t,y)
sifang(t,y,c1,t4,t3,t2,s4,s3,s2,ar2,n2,c2,ac3,ar3,n3,c41,c42,c43,c44),[0,200],[0.3,0.3,0.2,0.3]);
points=1:1:length(t);
```

```
plot(t,y(:,1),'r^-','linewidth',1,'markersize',3,'markerfacecolor','r','markerindices',points); hold on
plot(t,y(:,2),'b-','linewidth',1);
hold on
plot(t,y(:,3),'y-.','linewidth',1);
hold on
plot(t,y(:,4),'g--','linewidth',1);
hold on
set(gca,'XTick',[0:50:200],'YTick',[0.0:0.2:1.0])
set(gca,'YTickLabel',num2str(get(gca,'YTick')','%.1f')); axis([0
200 -0.05  1.05])
xlabel('$t$','interpreter','latex');
ylabel('Proportion');
zhuti=title('$x=0.3$'); set(zhuti,'interpreter','latex')
legend(Government regulatory agencies({\it\fontname{BodoniMT}x})','Bank
({\it\fontname{BodoniMT}y})','Non-bank financial institutions({\it\fontname{Bodoni MT}z})','High
-carbon enterprises({\it\fontname{Bodoni MT}w})');

c1=4,t4=5,t3=4,t2=3,s4=5,s3=4,s2=3,ar2=10,n2=3,c2=1,ac3=2,ar3=10,n3=2,c41=10,c42=4,c43=3,c4
4=2;
%subplot(3,1,1)
set(0,'defaultfigurecolor','w')
[t,y]=ode45(@(t,y)
sifang(t,y,c1,t4,t3,t2,s4,s3,s2,ar2,n2,c2,ac3,ar3,n3,c41,c42,c43,c44),[0,200],[0.4,0.3,0.2,0.3]);
points=1:1:length(t);
plot(t,y(:,1),'r^-','linewidth',1,'markersize',3,'markerfacecolor','r','markerindices',points); hold on
plot(t,y(:,2),'b-','linewidth',1);
hold on
plot(t,y(:,3),'y-.','linewidth',1);
hold on
plot(t,y(:,4),'g--','linewidth',1);
hold on
set(gca,'XTick',[0:50:200],'YTick',[0.0:0.2:1.0])
set(gca,'YTickLabel',num2str(get(gca,'YTick')','%.1f')); axis([0
200 -0.05  1.05])
xlabel('$t$','interpreter','latex');
ylabel('Proportion');
zhuti=title('$x=0.9$'); set(zhuti,'interpreter','latex')
legend(Government regulatory agencies({\it\fontname{BodoniMT}x})','Bank
({\it\fontname{BodoniMT}y})','Non-bank financial institutions({\it\fontname{Bodoni MT}z})','High
-carbon enterprises({\it\fontname{Bodoni MT}w})');
```

Figure(14)

```
clc;clear;
```

```
c1=4,t4=5,t3=4,t2=3,s4=5,s3=4,s2=3,ar2=10,n2=3,c2=1,ac3=2,ar3=10,n3=2,c41=10,c42
=4,c43=3,c44=2;
%subplot(2,1,1)
for i=0
    for j=0.1:0.2:1
        fork=0.1:0.2:1
            forl=0.1:0.2:1
            [t,y]=ode45(@(t,y)
sifang(t,y,c1,t4,t3,t2,s4,s3,s2,ar2,n2,c2,ac3,ar3,n3,c41,c42,c43,c44),[0 50],[i j k l]);
            grid on
            plot3(y(:,1),y(:,2),y(:,4),'linewidth',1);
            set(gca,'XTick',[0:0.2:1],'YTick',[0:0.2:1],'ZTick',[0:0.2:1])
            set(gca,'XTickLabel',num2str(get(gca,'XTick')','%.1f'));
            set(gca,'YTickLabel',num2str(get(gca,'YTick')','%.1f'));
            set(gca,'ZTickLabel',num2str(get(gca,'ZTick')','%.1f'));
            hold on
            axis([0 1 0 1 0 1])
            end
        end
    end
end
xlabel('$r$','interpreter','latex');
ylabel('$m$','interpreter','latex');
zlabel('$p$','interpreter','latex','Rotation',360);
title('x=0','interpreter','latex');
%%%%%%%%%%%%%%%%%%%%%%%%%
clc;clear;
c1=4,t4=5,t3=4,t2=3,s4=5,s3=4,s2=3,ar2=10,n2=3,c2=1,ac3=2,ar3=10,n3=2,c41=10,c42
=4,c43=3,c44=2;
%subplot(2,1,2)
for i=0. 8
    for j=0.1:0.2:1
        fork=0. 1:0.2:1
            forl=0.1:0.2:1
            [t,y]=ode45(@(t,y)
sifang(t,y,c1,t4,t3,t2,s4,s3,s2,ar2,n2,c2,ac3,ar3,n3,c41,c42,c43,c44),[0 50],[i j k l]);
            grid on
            plot3(y(:,1),y(:,2),y(:,4),'linewidth',1);
            set(gca,'XTick',[0:0.2:1],'YTick',[0:0.2:1],'ZTick',[0:0.2:1])
            set(gca,'XTickLabel',num2str(get(gca,'XTick')','%.1f'));
            set(gca,'YTickLabel',num2str(get(gca,'YTick')','%.1f'));
            set(gca,'ZTickLabel',num2str(get(gca,'ZTick')','%.1f'));
            hold on
            axis([0 1 0 1 0 1])
            end
        end
    end
end
xlabel('$r$','interpreter','latex');
ylabel('$m$','interpreter','latex');
zlabel('$p$','interpreter','latex','Rotation',360);
title('{\it\fontname{BodoniMT}x}=1');
```

The simulation tool used is Matlab2017a.The remaining simulation figures 6-15 were

obtained by modifying the relevant parameters based on the above program.
The specific parameter assignments are as follows:

To more intuitively reflect the evolutionary path of the four-game players of government regulatory authorities, bank financial institutions, non-bank financial institutions, and high-carbon enterprises, this study chooses to introduce the theory of willingness behavior into simulation analysis based on the stable point (0, 1, 1, 1). It examines the impact of the initial willingness of the game players and changes in relevant parameters on the stable state of the four players' strategy selection. Based on this, this article refers to the practices of Sun et al.[5] and Cui et al. [29] and assign the relevant parameters based on the parameter constraints of scenario five as follows: $c_1 = 4$, $\pi_2 = 3$, $\pi_3 = 4$, $\pi_4 = 5$, $s_2 = 3$, $s_3 = 4$, $s_4 = 5$, $\Delta r_2 = 10$, $\eta_2 = 3$, $c_2 = 1$, $\Delta c_3 = 2$, $\Delta r_3 = 10$, $\eta_3 = 2$, $c_{41} = 10$, $c_{42} = 4$, $c_{43} = 3$, $c_{44} = 2$ 。
